# Glycosaminoglycan modifications of betaglycan regulate ectodomain shedding to fine-tune TGF-β signaling responses in ovarian cancer

**DOI:** 10.1186/s12964-024-01496-y

**Published:** 2024-02-15

**Authors:** Alex S. Choi, Laura M. Jenkins-Lane, Wade Barton, Asha Kumari, Carly Lancaster, Calen Raulerson, Hao Ji, Diego Altomare, Mark D. Starr, Regina Whitaker, Rebecca Phaeton, Rebecca Arend, Michael Shtutman, Andrew B. Nixon, Nadine Hempel, Nam Y. Lee, Karthikeyan Mythreye

**Affiliations:** 1 Department of Pathology and O’Neal Comprehensive Cancer Center, Heersink School of Medicine, University of Alabama at Birmingham, Birmingham, AL 35233 USA; 2 Department of Chemistry and Biochemistry, University of South Carolina, Columbia, SC 29208 USA; 3 Department of Medicine and Duke Cancer Institute, Duke University Medical Center, Durham, NC 27710 USA; 4 Department of Obstetrics and Gynecology, Duke University Medical Center, Durham, NC USA; 5 Department of Medicine, Division of Hematology-Oncology, Hillman Cancer Center, University of Pittsburgh School of Medicine, Pittsburgh, PA 15213 USA; 6 Department of Obstetrics and Gynecology, and Microbiology and Immunology, College of Medicine, Pennsylvania State University, Hershey, PA 17033 USA; 7 Department of Gynecology Oncology, Heersink School of Medicine, University of Alabama School of Medicine, Birmingham, AL 35233 USA; 8 Division of Pharmacology, Chemistry and Biochemistry, College of Medicine, University of Arizona, Tucson, AZ 85721 USA; 9 Department of Drug Discovery & Biomedical Sciences, College of Pharmacy, University of South Carolina, Columbia, SC 29208 USA

**Keywords:** TβRIII, Betaglycan, Ectodomain shedding, Soluble-betaglycan, Cancer biology, Ovarian cancer, Cell signaling, Glycosaminoglycan, Transforming growth factor β (TGF-β)

## Abstract

In pathologies including cancer, aberrant Transforming Growth Factor-β (TGF-β) signaling exerts profound tumor intrinsic and extrinsic consequences. Intense clinical endeavors are underway to target this pathway. Central to the success of these interventions is pinpointing factors that decisively modulate the TGF-β responses. Betaglycan/type III TGF-β receptor (TβRIII), is an established co-receptor for the TGF-β superfamily known to bind directly to TGF-βs 1–3 and inhibin A/B. Betaglycan can be membrane-bound and also undergo ectodomain cleavage to produce soluble-betaglycan that can sequester its ligands. Its extracellular domain undergoes heparan sulfate and chondroitin sulfate glycosaminoglycan modifications, transforming betaglycan into a proteoglycan. We report the unexpected discovery that the heparan sulfate glycosaminoglycan chains on betaglycan are critical for the ectodomain shedding. In the absence of such glycosaminoglycan chains betaglycan is not shed, a feature indispensable for the ability of betaglycan to suppress TGF-β signaling and the cells' responses to exogenous TGF-β ligands. Using unbiased transcriptomics, we identified TIMP3 as a key inhibitor of betaglycan shedding thereby influencing TGF-β signaling. Our results bear significant clinical relevance as modified betaglycan is present in the ascites of patients with ovarian cancer and can serve as a marker for predicting patient outcomes and TGF-β signaling responses. These studies are the first to demonstrate a unique reliance on the glycosaminoglycan chains of betaglycan for shedding and influence on TGF-β signaling responses. Dysregulated shedding of TGF-β receptors plays a vital role in determining the response and availability of TGF-βs’, which is crucial for prognostic predictions and understanding of TGF-β signaling dynamics.

**Supplementary Information:**

The online version contains supplementary material available at 10.1186/s12964-024-01496-y.

## Background

Type III TGF-β receptor (TβRIII) / betaglycan (BG) is a widely expressed transmembrane proteoglycan and an established coreceptor for a subset of the TGF-β superfamily of ligands [1, 2]. As a co-receptor, BG can either increase or decrease signaling by TGF-β superfamily members that directly bind BG including all isoforms of TGF-β1,2 and 3 [3, 4], as well as BMP2, 4, 7 [5, 6], GDF-5 [6], Inhibin A [7], and Inhibin B [8]. Betaglycan binds TGF-β2 with greater affinity than TGF-β1 or TGF-β3 [9] and thus cells lacking BG expression do not respond as well to TGF-β2 as compared to TGF-β1 and TGF-β3 in equimolar settings, requiring up to 500-fold higher concentrations of TGF-β2 to achieve the same potency of activation as TGF-β1 and TGF-β3 in the absence of BG [10–12]. These observations are not cell-line specific and have been reported in multiple cell types including in cancer cell lines and non-oncogenic models [2, 9, 13]. BG likely functions to bind TGF-β2 and concentrate the ligand to facilitate access to the Type II-TGF-β receptor kinase, effectively restoring cellular sensitivity to TGF-β2 to comparable levels of TGF-β1/3 [3]. Both BG and TGF-β2 knockout mice show similar defects in vivo [14, 15], demonstrating the physiological reliance of TGF-β2 on BG. In the case of TGF-β1 responsiveness, BG can either stimulate or inhibit TGF-β1 signaling [16–19].

Transmembrane proteoglycans including betaglycan can be proteolytically cleaved, releasing soluble ectodomain into the ECM in a process called ectodomain shedding [20]. Only 2 to 4% of cell surface molecules undergo shedding [21, 22], and dysregulated shedding is associated with various pathologies including cancer [20, 22, 23] suggesting that maintaining the levels of shed proteins may be critical. Notably, in women’s cancers including ovarian [24], breast [17], as well as granulosa cell tumors that arise from ovarian sex cord-stromal cells [25], lower BG expression in the tumor cells as compared to adjacent normal tissue has been reported [13, 17, 24–26], with lower BG expression found to be an indicator of poor patient outcomes [24, 27]. Previous studies also indicate that compared to membrane-bound BG, shed-BG can reduce TGF-β signaling in a concentration-dependent manner [19]. In tumor cells, shed-BG reduced cell migration, invasion, and metastatic properties by limiting TGF-β available at the cell surface, causing a reduction in TGF-β signaling [17, 19, 28]. Similar observations have been made in normal epithelial cells as well [29].

General regulators of proteoglycan shedding include proteases (also called sheddases) of the ADAM/ADAM-related family of proteins [30, 31], MMPs, most notably, but not limited to MMPs-1,2, and 7 [30, 32–35], as well as chemical agents such as phorbol ester and calcium ionophores [22, 33] and specific serum factors [20, 22, 23, 33, 36]. BG shedding is however unaffected by phorbol esters, calcium ionophores, PMA, and serum factors [21, 37] but is stimulated by pervanadate [33] and is inhibited by TAPI-2, an MT-MMP/ADAM protease inhibitor [38].

A distinctive feature of BG lies in the extracellular domain modifications with glycosaminoglycan (GAG) chains at serine residues Ser^534^ and Ser^545^, to which heparan sulfate (HS) and chondroitin sulfate (CS) chains are covalently attached [39–44] . HS is a repeating unit of N-acetylglucosamine and glucuronic acid and CS is a repeat of n-acetyl-galactosamine and glucuronic acid [45]. BG is commonly referred to as a “part-time proteoglycan” since BG can be expressed on the cell surface with or without GAG chains [46–49]. Previous reports suggest that GAG chains are not essential for the ligands that bind to the core domain of BG [9, 39]. However, GAG chains of BG can mediate the binding of growth factors such as FGF2 [50], and Wnt3A [44], where Wnt3A signaling can be influenced by the HS and CS chains [44]. In addition to affinities of ligands to the GAG chains of BG, the presence of the GAG chains has also been proposed to prevent access of the BG core binding ligands to their respective signaling receptors, suggesting disruption of TGF-β signaling and function [43, 51].   

While previous studies have demonstrated the effect of overall BG levels on TGF-β signaling, a thorough understanding of the role of BG GAG chains and their influence on regulating TGF-β signaling if any, is currently lacking. Here, we sought to address this. We report a direct role for the BG GAG modifications on ectodomain shedding of BG and identify BG modification-specific expression changes in TIMP3, a negative regulator of BG ectodomain shedding. We also demonstrate that the presence of GAG modifications on BG is critical for fine-tuning TGF-β signaling and invasive properties of tumor cells. Lastly, we report that higher amounts of shed-BG in the ascites fluid of ovarian cancer patients correlate with advanced-stage disease and serve as a negative predictor of patient survival.

## Materials and methods

### Cell Lines and Reagents

#### Ovarian epithelial carcinoma cell lines

HEYA8, SKOV-3, and OVCA-429 were obtained from the Duke Gynecology/Oncology Bank (Durham, NC) and ATCC (ATCC® HTB-77™). CHO-K1 epithelial cell lines pgsA-745 (ATCC® CRL-2242™), and pgsD-677 (ATCC® CRL-2244™) were obtained from ATCC (Manassas, VA). HEYA8, SKOV-3, and OVCA-429 were cultured in RPMI 1640 (ATCC® 30-2001ATCC™) containing L-glutamine, 10% FBS, and 100 units of penicillin–streptomycin. CHO-K1 cell lines pgsA-745 and pgsD-677 were cultured in DMEM/F-12 medium (ATCC 30–2006) DMEM: F-12 Medium contains 2.5 mM L-glutamine, 15 mM HEPES, 0.5 mM sodium pyruvate, and 1200 mg/L sodium bicarbonate, 10% FBS, and 100 units of penicillin–streptomycin. All cell lines were maintained at 37°C in a humidified incubator at 5% CO_2_, routinely checked for mycoplasma, and experiments were conducted within 3–10 passages depending on the cell line. Cell line authentication was performed at the Heflin Center for Genomic Science Core Laboratories at UAB.

#### Antibodies

Human TGF-beta RIII (catalog AF-242) was purchased from R&D Biosystems (Minneapolis, MN), Phospho-SMAD2 (Ser465/467)/SMAD3 (Ser423/425) (catalog #8828), Smad2/3 (D7G7) (catalog #8685), Smad2 (D43B4) XP® Rabbit mAb (catalog #5339), TIMP3 (D74B10) Rabbit mAb (catalog #5673) were obtained from Cell Signal Technology (Danvers, MA). Actin antibody (C-2) (catalog sc-8432) was obtained from Santa Cruz Biotech (Santa Cruz, CA). Rabbit anti TGFBR1 (pS165) (catalog #620–910) antibody was obtained from Abbomax, Inc. (San Jose, CA).

#### Other reagents

[^125^ I]-Bolton-Hunter labeled Transforming Growth Factor- β1 (Human, Recombinant) (catalog NEX267) was obtained from Perkin Elmer (Waltham, MA). This product was however discontinued in 2019. Human TGF-beta RIII DuoSet ELISA (catalog DY242), DuoSet ELISA Ancillary Kit (catalog DY008), Recombinant Human TGF-beta RIII Protein (catalog 242-R3), Heparinase III (catalog 6145-GH-010) and Chondroitinase ABC (catalog No. C3667) were obtained from Sigma-Aldrich, and recombinant TGF-β1, and TGF-β2, were purchased from R&D Systems. A83-01 (catalog 100–1041, 72,024) was purchased from Stemcell Technologies.

### Plasmid constructs, generation of stable and transient expression cell lines

#### Constructs

Betaglycan (BG) constructs were generated as described previously [17, 26, 43, 44, 50, 52]. FL-BG consists of HA-tagged human BG in pcDNA 3.1( +) [43, 53]. The BG-ΔGAG construct was generated by introducing double ser-ala point mutation at amino acids 534 and 545 to prevent GAG modifications [53–56]. Single GAG attachment site mutants S534A (CS-BG), and S545A (HS-BG) constructs were all generated by site-directed mutagenesis (Agilent Technologies 210,515). Constructs were confirmed by sequencing.

#### Stable cell lines

FL-BG, ∆GAG-BG, S534A (CS-BG), and S545A (HS-BG) constructs were cloned into a pHIV-dTomato lentiviral backbone (Plasmid #21,374, Addgene, Cambridge, MA) by the Center for Targeted Therapeutics Core Facility at the University of South Carolina (Columbia, SC) followed by lentiviral particle generation. Infected cells were sorted by dTomato expression at the flow cytometry core at the University of South Carolin Flow Cytometry Core Facility or UAB Flow Cytometry and Single-Cell Core facility.

 Betaglycan CRISPR knockout cell line was generated using Origene CRISPR/CAS9 genome-wide knockout kit (GE100021). 10 MOI of CRISPR BG pCAS guide virus and 8µg/mL of polybrene were used for each infection. Puromycin selection was performed on the infected cells. Surviving cells with puromycin resistance were then isolated into a monoclonal population using a limited dilution method. Wells containing a single colony of cells were then expanded and characterized by [^125^ I]-TGF-β1 binding and crosslinking followed by immunoprecipitation using BG antibody. KO clones with no BG expression were chosen for further experiments. All KO cells were maintained with 0.5µg/mL of puromycin.

#### Transient cell lines

Adenoviral constructs for FL-BG, and ΔGAG-BG were used to transduce cells at MOI of 50 to 200 IFUs/cell, and infections were performed as previously described [49, 50, 57]. Origene siRNA-27 kit was used for transient knockdown of TIMP3 expression (SR304839 Locus IF 7078). Cells were reverse-transfected using Lipofectamine-RNAiMAX with 10 nM of universal negative control RNA duplex (Scrambled) compared to the cell transfected with 10 nM of pooled duplexes targeting TIMP3. siRNA/Scramble vectors were transfected into HEYA8 and SKOV-3 cells using RNAiMAX. Media was refreshed and samples were collected 48–72 h post-transfection.

### Immunoprecipitation, Western Blotting, and Immunofluorescence

Immunoprecipitation and Western blotting were performed using standard techniques [44, 57, 58]. For immunoprecipitations of shed-BG and membrane-bound BG, conditioned media/cell lysed in CO-IP lysis buffer (50 mM Tris–HCl, pH 7.5, 150 mM of NaCl, 1% Nonidet P-40, 10% glycerol, 1 mM DTT, 25 mM NaF, 1 mM Na_3_VO_4_ and 1 × protease inhibitor mixture (catalog No. P8340, Sigma-Aldrich)) was incubated overnight with 2.5µg of anti-human BG antibody and 30µL of Protein G-Sepharose beads in 4°C with mild agitation. The next day, PGS beads were washed three times with cold PBS and re-suspended in the 2 × Laemmli sample buffer. For immunofluorescence, HEYA8, Control-Vector, FL-BG, and ∆GAG-BG expressing cells were seeded onto coverslips in 12-well plates at a density of 5 × 10^4^ cells/well. After 24 h, cells were serum starved overnight and then treated with 25 pM TGF-β1/2 for 1 hr unless otherwise indicated. Cells were fixed in 4% paraformaldehyde and permeabilized with 0.1% TritonX-100, followed by blocking with 5% BSA in PBS for 1 hr. SMAD2 was labeled using the D43B4 CST SMAD2 antibody and incubated with an Alexa-conjugated secondary antibody (Molecular Probes, Eugene, OR). Nuclei were stained with Hoechst 33258. Coverslips were mounted using ProLong Gold Antiface mountant (Thermo Fisher catalog P36930). Immunofluorescence imaging was captured using an EVOS M7000 microscope at 60 × magnification. SMAD2 localization was quantified using a Cell Profiler [59] pipeline.

### Crosslinking and Binding with [^125^ I]-TGF-β1

Cell surface receptors and conditioned media [^125^ I]-TGF-β1 crosslinking and binding methods have been extensively described previously [2, 39, 60]. Cell surface receptor binding was conducted in a cold room to inhibit receptor internalization. For cell surface labeling, 100 pM of [^125^ I]-TGF-β1 in HEPES-KRH Buffer was used (Final concentration. NaCl, 116 mM. KCl, 4 mM. MgCl2, 1 mM. CaCl2, 1.8 mM. Glucose, 25 mM. HEPES acid, 10 mM. Adjust pH to 7.4). For shed-BG binding, conditioned media from BG GAG mutant expressing cells were incubated in full-serum media or serum-free media, as noted in the figure legends, and were directly labeled with 200 pM of [^125^ I]-TGF-β1. Washing steps were omitted in conditioned media BG binding. Cell lysate samples were lysed with 2 × Laemmli sample buffer or CO-IP buffer, followed by the immunoprecipitation protocol described above. SDS-page gels were dried onto a filter paper at 80°C for 2.5 hrs on a gel dryer. Dried gels on filter papers were developed onto a phosphor screen for 10 – 21 days. Imaging of the phosphor screen was performed on the GE Typhoon system. The scanned image was then analyzed using ImageQuant software.

### RNA isolation and semi-quantitative RT-PCR

Total RNA was isolated using TRIzol reagent/protocol from Invitrogen. RNA was reverse transcribed using iScript Reverse Transcription Supermix and iTaq Universal SYBR Green Supermix. Expression data were normalized to the geometric mean of housekeeping genes *RPL13A* and *HPRT1.* The qRT-PCR primers sequences used were: *RPL13A* forward: AGATGGCGGAGGTGCAG; reverse GGCCCAGCAGTACCTGTTTA, *HPRT1* forward: TGACCTTGATTTATTTTGCATACC; reverse: CGAGCAAGACGTTCAGTCCT, *TGFBR3* forward: CGTCAGGAGGCACACACTTA; reverse: CACATTTGACAGACAGGGCAAT, and *TIMP3* forward: GTGGTCAGCCTCTCTCACAC; reverse: AAGACCCTTCTTTGCCCAGG.

### ELISA

Betaglycan ELISA (DY242) from R&D systems was utilized for the majority of this study apart from the processing of Duke repository AF samples, the methodology for which is as described previously [19, 29] and performed according to the manufacturer’s instructions to quantitatively measure BG concentration in conditioned media. Conditioned media was collected in full-serum media or serum-free media for durations noted in the figure legends. Biological duplicates of samples were collected per condition and 2–3 technical replicates per biological replicate were analyzed. The optical density of each well was measured via a Gen-5 plate reader set to 450 nm with wavelength correction set to 540 nm. Optical density values were used to calculate the concentration of sBG using a 4PL calculator (AAT-BIO) based on recombinant human BG standard values ran with every set of experiments.

### Trans-well invasion assay

2 x 10^4^ HEYA8/SKOV-3 and 1 x 10^5^ SKOV-3 BGKO cells were plated on a matrigel-coated (400 μg/mL) 8 μm trans-well filter in serum-free media. 10% FBS media was used as a chemoattractant in the bottom chamber. Apical cells were scraped off and invaded cells were fixed and stained using a Three-Step stain set from Thermo Fisher. 3–5 random images were taken per filter using a 10X objective on the EVOS M7000 microscope. Cells were counted using the ImageJ Cell-counter plugin.

### Patient Ascites

Specimens from patients diagnosed with primary ovarian cancer were collected and banked after informed consent at Duke University Medical Center, Pennsylvania State University College of Medicine (Hershey, PA), or the University of Alabama Birmingham, with approval for the study grant from the Duke University’s institutional research ethics board, Penn State College of Medicine and UAB Institutional Review Boards (IRB) respectively. All samples were previously banked frozen samples. Acellular ascites fluid was briefly spun down at 1200 rpm and then the supernatant was collected. The protein concentration of each AF sample was measured using a Pierce BCA Protein Assay kit (Thermo scientific 23227) and then normalized to equal concentrations for downstream analysis.

### RNA-SEQ

RNA libraries were prepared using NEB Next Ultra II RNA Library prep kit following manufacturers protocols. Quality check of indexed sequences was performed using FastQC and then trimmed using Trim Galore-0.4.5. Read counts of annotated genes were obtained using feature count. Differential expression analysis was performed using the bioJupies web tool [61]. RNA-seq data have been deposited in the NCBI Gene Expression Omnibus (GEO) database (Accession number GSE237403).

### Statistics

All graphs are representative of 3–5 independent biological experiments with individual points denoting the average of each experiment unless described otherwise in figure legends. Data are expressed as Mean ± SEM. Statistical analyses were performed using GraphPad Prism 11, described in the figure legends. The difference between the two groups was assessed using a two-tailed *t*-test. Multiple group comparisons were carried out by the analysis of variance (ANOVA). Survival analysis and correlation analysis were performed using GraphPad Prism 11 using the Mantel-Cox test, and Spearman correlation test.

## Results

### TβRIII / betaglycan (BG) glycosaminoglycan modifications promote ectodomain shedding

To address whether glycosaminoglycan modifications on BG impact ectodomain shedding, we used a previously generated double Ser- Ala point mutation at S534 and S545 of BG, to eliminate the GAG chain attachment sites on BG [39–44]. Expression of this construct has been reported to prevent GAG attachment [33, 39, 50, 54] and was recapitulated in cancer cells used here that represent a spectrum of epithelial ovarian cancers (Suppl. Figure 1A).

**Fig. 1:**
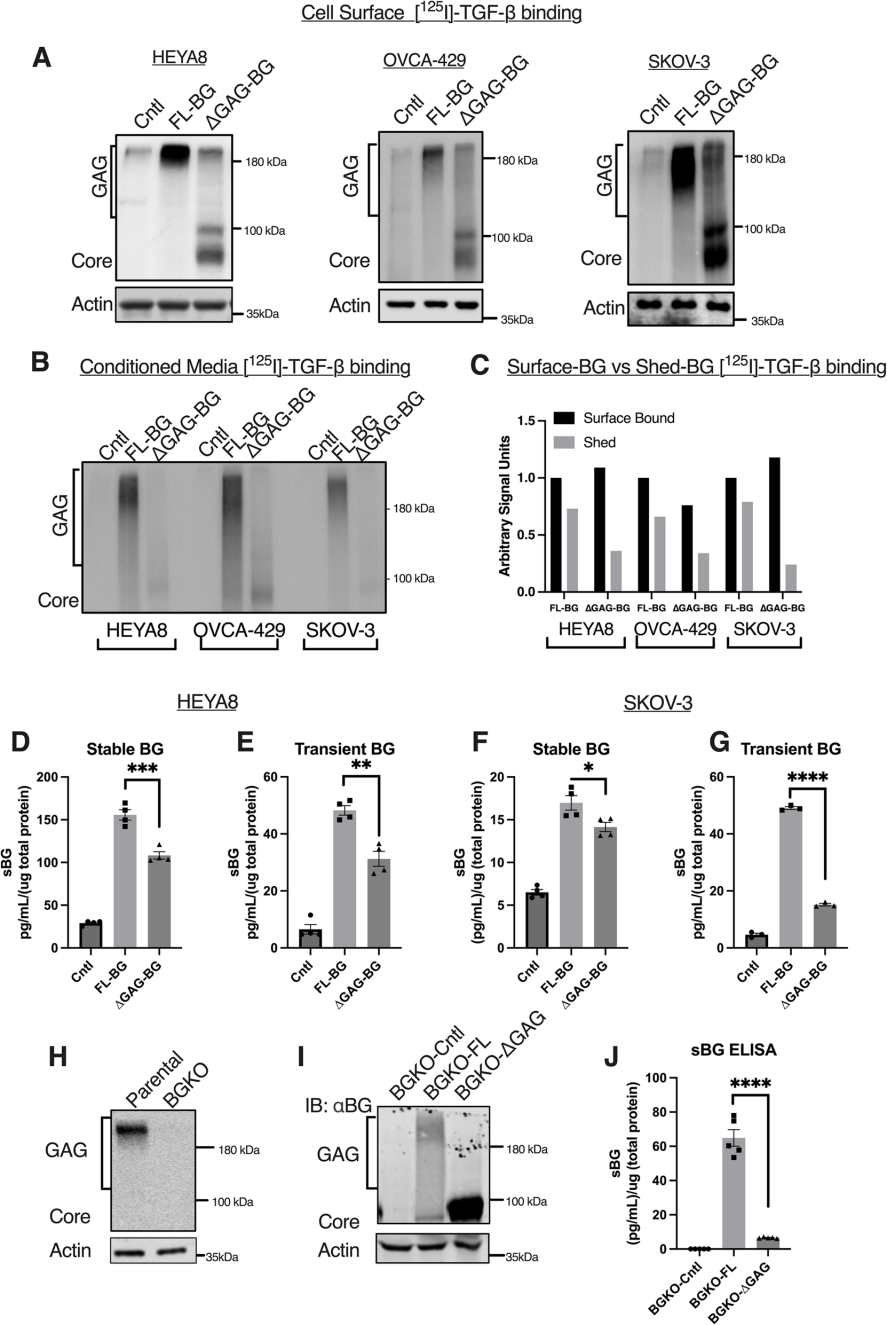
Glycosaminoglycan-modified betaglycan (BG) sheds more than unmodified BG. **A**, **B** Autoradiograph of samples after [^125^ I]-TGF-β1 binding and crosslinking of either **A** total cell lysates or **B** from conditioned media of indicated cells with stable (HEYA8, SKOV-3) or transient (OVCA-429) expression of BG (control-vector, FL-BG, ∆GAG-BG) and **C** quantification of the cell surface binding autoradiograph to the conditioned media binding autoradiograph of indicated cells expressing FL-BG or ∆GAG-BG. **D-G** BG-ELISA from the conditioned media of indicated HEYA8 **D**, **E** and SKOV-3 **F**, **G** cells. The concentration of shed-BG normalized to the total protein concentration of the cells is plotted by Mean ± SEM, (*n* = 4 for stable and *n* = 3 for transient). *p < 0.05; **p < 0.01; ***p < 0.001; ****p < 0.0001, One-way ANOVA followed by unpaired t-test between FL-BG and ∆GAG-BG (HEYA8 stable, *p* = 0.0007, transient, *p* = 0.0018, FL-BG vs ∆GAG-BG) (SKOV-3 stable, *p* = 0.034, transient, p < 0.0001, FL-BG vs ∆GAG-BG). **H** Autoradiograph of SKOV-3 parental and BG CRISPR Knockout (BGKO) cells radiolabeled with [^125^ I]-TGF-β1 followed by immunoprecipitation using anti-BG antibody. **I** Western blot of SKOV-3 BGKO cells transiently expressing FL-BG (BGKO-FL) or ∆GAG-BG (BGKO-∆GAG) immunoblotted with anti-BG. **J** BG ELISA of conditioned media collected from the SKOV-3 BGKO cells expressing FL-BG (BGKO-FL) and ∆GAG-BG (BGKO-∆GAG). The concentration of shed-BG normalized to the total protein concentration of the cells is plotted (Mean ± SEM, *n* = 5). ****p < 0.0001, One-way ANOVA followed by unpaired t-test between BGKO-FL and BGKO-∆GAG

HEYA8, SKOV-3, and OVCA-429 ovarian cancer (OVCA) cell lines were chosen due to low endogenous expression of BG (Suppl. Figure 1B) and BG constructs (Full-Length (BG-FL) or BG lacking GAG chains (BG-∆GAG) were either transiently or stably overexpressed (Fig. 1A**, **Suppl. Figure 1A). Previous studies have reported no effects of the GAG modifications on BG on its ability to bind TGF-β [39, 43] and this was confirmed here as well using cell surface receptor [^125^ I]-TGF-β1 binding and crosslinking (Fig. 1A). Cell surface binding of BG to TGF-β confirmed that FL-BG cells present GAG modifications ranging from ~ 90 kDa to 250 kDa (Fig. 1A second lanes), while ΔGAG-BG expressing cells which are competent at binding TGF-β showed BG devoid of high molecular weight GAG chains with a core band at ~ 95 kDa (Fig. 1A third lanes).

To test the amount of shed BG as a result of cells expressing either FL-BG or ∆GAG-BG; conditioned media was subjected to [^125^ I]-TGF-β1 binding and crosslinking followed by immunoprecipitation using an anti-BG antibody to assess shed-BG. Shed-BG with glycosylated forms ranging from 90 to 250 kDa were present in the conditioned media of FL-BG-expressing cells as previously reported [19, 33, 38] and seen here (Fig. 1B**,** FL-BG labeled lanes). However, media from ΔGAG-BG expressing cells had reduced [^125^ I]-TGF-β1 intensity compared to the cell surface [^125^ I]-TGF-β1 bound to ΔGAG-BG (Fig. 1B ∆GAG-BG labeled lanes compared to Fig. 1A (cell-surface BG [^125^ I]-TGF-β1 binding). Quantification of [^125^ I]-TGF-β1 surface-bound BG and shed-BG signal units in conditioned media samples indicate that ΔGAG-BG expressing cells had less than half the levels of shed-BG compared to FL-BG expressing cells in all cell lines. (Fig. 1C).

To quantitively assess this difference in BG ectodomain shedding of FL-BG and ∆GAG-BG expressing cells, and to rule out differences in TGF-β binding in the media we used a BG ELISA to quantify differences in the amount of shed/soluble BG in two cell lines with low BG expression and either transiently or stably expressing FL and ∆GAG mutants (HEYA8 Figs. 1D-E and SKOV-3 Figs. 1F-G). We find that FL-BG expressing HEYA8 and SKOV-3 cells showed significantly higher shed-BG as compared to ΔGAG-BG expressing HEYA8 and SKOV-3 cells regardless of whether BG was expressed stably or transiently (Figs. 1D-G). In addition, to account for any endogenous expression of BG that may interfere with the shedding differences seen, we generated a CRISPR knockout of BG in SKOV-3 cells (BGKO, Fig. 1H, Suppl. Figures 1B, lane 3). Knockout cells were restored with either FL-BG or ΔGAG-BG resulting in BGKO-FL or BGKO-ΔGAG cells (Fig. 1I). We find that BGKO-ΔGAG cells shed only 1/10th as much as the BGKO-FL cells (65 pg/mL/(µg total protein) compared to 6 pg/mL/(µg total protein) in ΔGAG-BG expressing cells (Fig. 1J). Together, these data in overexpression and knockout backgrounds demonstrate unequivocally, quantitative differences in the shedding of fully modified betaglycan as compared to unmodified BG.

### Heparan sulfate glycosaminoglycan chains preferentially enhance BG shedding

BG can present both HS and CS modified glycosaminoglycan chains [18, 19, 21, 23] in ovarian cancer cell lines as confirmed by enzymatic digestion using Chondroitinase ABC and Heparinase III (Suppl. Figure 2).

**Fig. 2:**
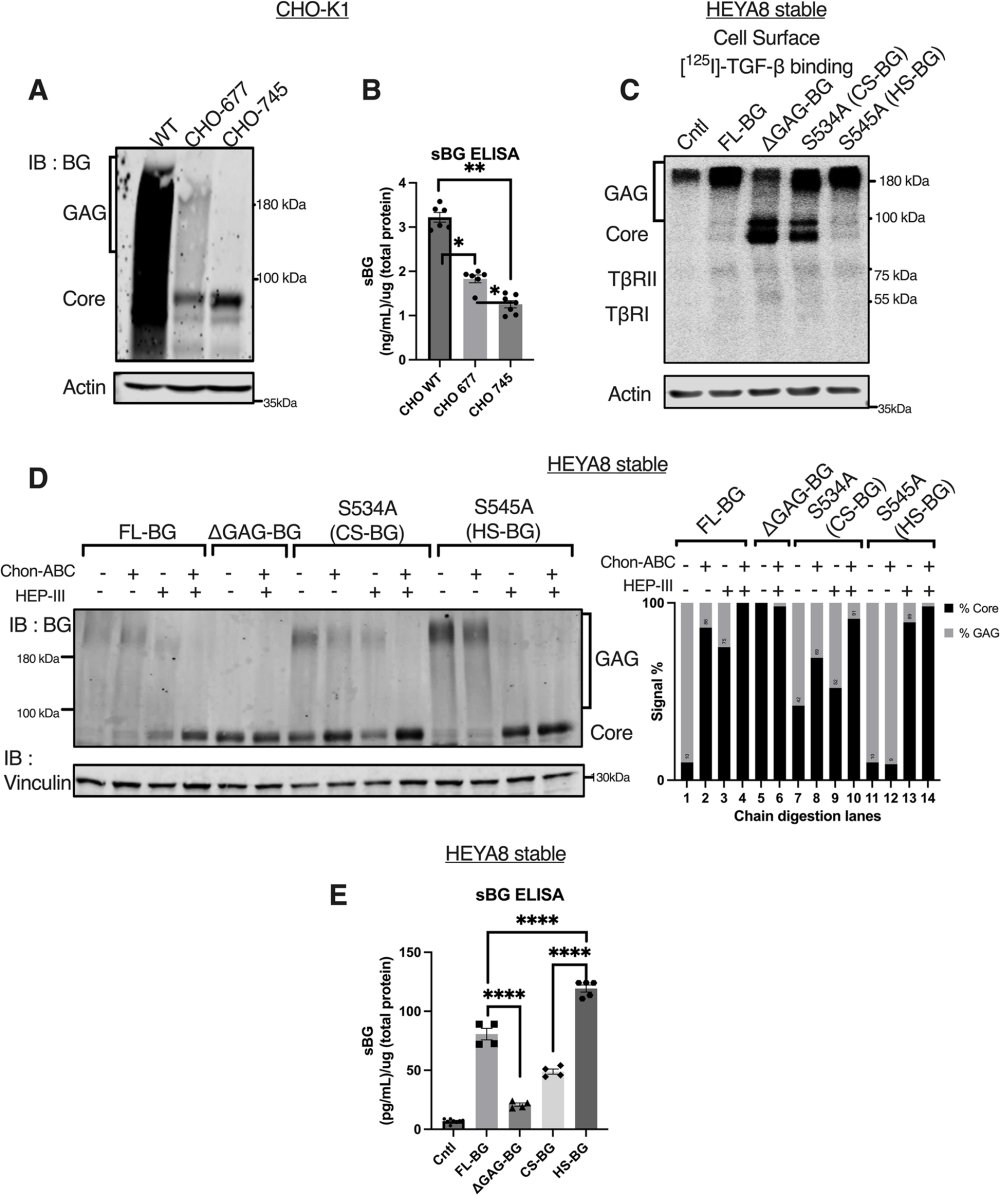
Heparan sulfate modifications of BG promote ectodomain shedding. **A** Western blot of BG in CHO-K1 WT, 677, and 745 cells expressing FL-BG. **B** ELISA of BG from conditioned media collected from CHO-K1 WT, 677, and 745 cells expressing FL-BG. The concentration of shed-BG normalized to the total protein concentration of the cells is plotted by Mean ± SEM, (*n* = 6) *p < 0.05, **p < 0.005; One-way ANOVA followed by unpaired t-tests between CHO WT vs CHO 677, CHO WT vs CHO 745, and CHO 677 vs CHO 745 (*p* = 0.039 WT vs 677, *p* = 0.0018 WT vs. 745, p = 0.0456, 677 vs. 745). **C** Autoradiograph of samples after [^125^ I]-TGF-β1 binding and crosslinking of cell surface receptors followed by immunoprecipitation using anti-BG antibody in indicated cells. **D** Western blot of BG from indicated HEYA8 cells after enzymatic treatment using chondroitinase-ABC (0.4U) and/or heparinase-III (50ng/mL) and quantification of western blot. The signal intensity of the BG-core band at 90 kDa was compared to the intensity of the larger GAG-modified BG band intensity spanning 100kDA to 250 kDa. Graph is plotted as %GAG to % core signal intensity normalized to the actin levels. **E** BG ELISA of the conditioned media collected from indicated HEYA8 cells. The concentration of shed-BG normalized to the total protein concentration of the cells is plotted by Mean ± SEM, (*n* = independent trials for Cntl = 8, FL-BG = 4, ∆GAG-BG = 4, S534A (CS-BG) = 4, S545A (HS-BG) = 5).; ****p < 0.0001, One-way ANOVA followed by unpaired t-test. *p* =  < 0.0001, FL-BG vs ∆GAG-BG, *p* < 0.0001, CS-BG vs. HS-BG, *p* = 0.0002, FL-BG vs. HS-BG)

To assess whether HS and/or CS modified chains differentially impact shedding, we first utilized CHO-K1 as control (wild type), and CHO-677 cells that are devoid of N-acetylglucosaminyltransferase and glucuronosyltransferase activities leading to the absence of HS GAG modifications [62] and CHO-745 mutant cells that are xylosyltransferase deficient leading to the absence of both HS and CS modifications [63]. BG-FL expressed in CHO-K1 WT, 677, and 745 cells respectively presents modifications as anticipated (Fig. 2A) and as described previously [44]. Analysis of conditioned media from the CHO cells for soluble BG by ELISA revealed that WT CHO cells expressing fully GAG-modified BG shed twice as much as the single CS chain expressing 677 cells (WT = 3.2 ng/mL/(µg total protein) vs. 677 = 1.83 ng/mL/(µg total protein) (Fig. 2B). The greatest reduction was seen upon eliminating HS chains (CHO 745 cells) as compared to WT BG expressing cells (3.2 ng/mL/(µg total protein) to 1.2 ng/mL/(µg total protein) (Fig. 2B). While a further small reduction in shedding was seen in CHO 745 cells that lacked all modifications (Fig. 2A, B third lane), the differences between BG in CHO745 and CHO 677 were statistically significant, suggesting that eliminating HS modifications was likely the largest contributor to the shedding reduction of BG.

To specifically test the effects of such HS modified GAG chains on BG further, we generated HEYA8 stable cell lines expressing BG with either predominantly HS GAG chains or predominantly CS forms of GAG chains (Fig. 2C, D). This was accomplished by generating single point mutations of S545A or S534A followed by expression of the mutants in HEYA8 cells. S534A was previously reported to be modified by both CS and HS with a preference for CS modifications [39, 41, 60] and S545 was previously reported to be modified primarily by HS GAG chains [2, 39, 44, 47]. [^125^ I]-TGF-β1 cell surface binding and crosslinking confirmed the cell surface availability and TGF-β binding capability of the BG mutants (Fig. 2C). Chondroitinase ABC and Heparinase III digestion of BG GAG chains confirmed the presence of both CS and HS modified GAG chains on S534A (Fig. 2D lanes 7–10, signal % quantification lanes 7–10) with complete loss of modifications in S545A after heparinase III digestion (Fig. 2D, lane 13, signal % quantification lane 13). BG ELISA of the shed BG (sBG) in the conditioned media of the BG-expressing cells revealed that cells expressing HS only (S545A) BG shed 1.5 times more than cells expressing FL BG (119 pg/mL versus 80 pg/mL respective) (Fig. 2E) and 2.5 times higher than cells expressing S534A (CS modifications alone 49 pg/mL versus S545A-HS BG at 119 pg/mL) (Fig. 2E). The least amount of shedding was seen in the ∆GAG cells as seen in Fig. 1 and Fig. 2E. The use of the individual mutants along with the CHO cell lines together indicates that HS modifications on BG facilitate shedding to a greater extent than CS modifications with a significant reduction seen upon loss of both modifications.

### GAG chains on BG are critical for fine-tuning TGF-β cellular signaling and cell migration responses

**Fig. 3:**
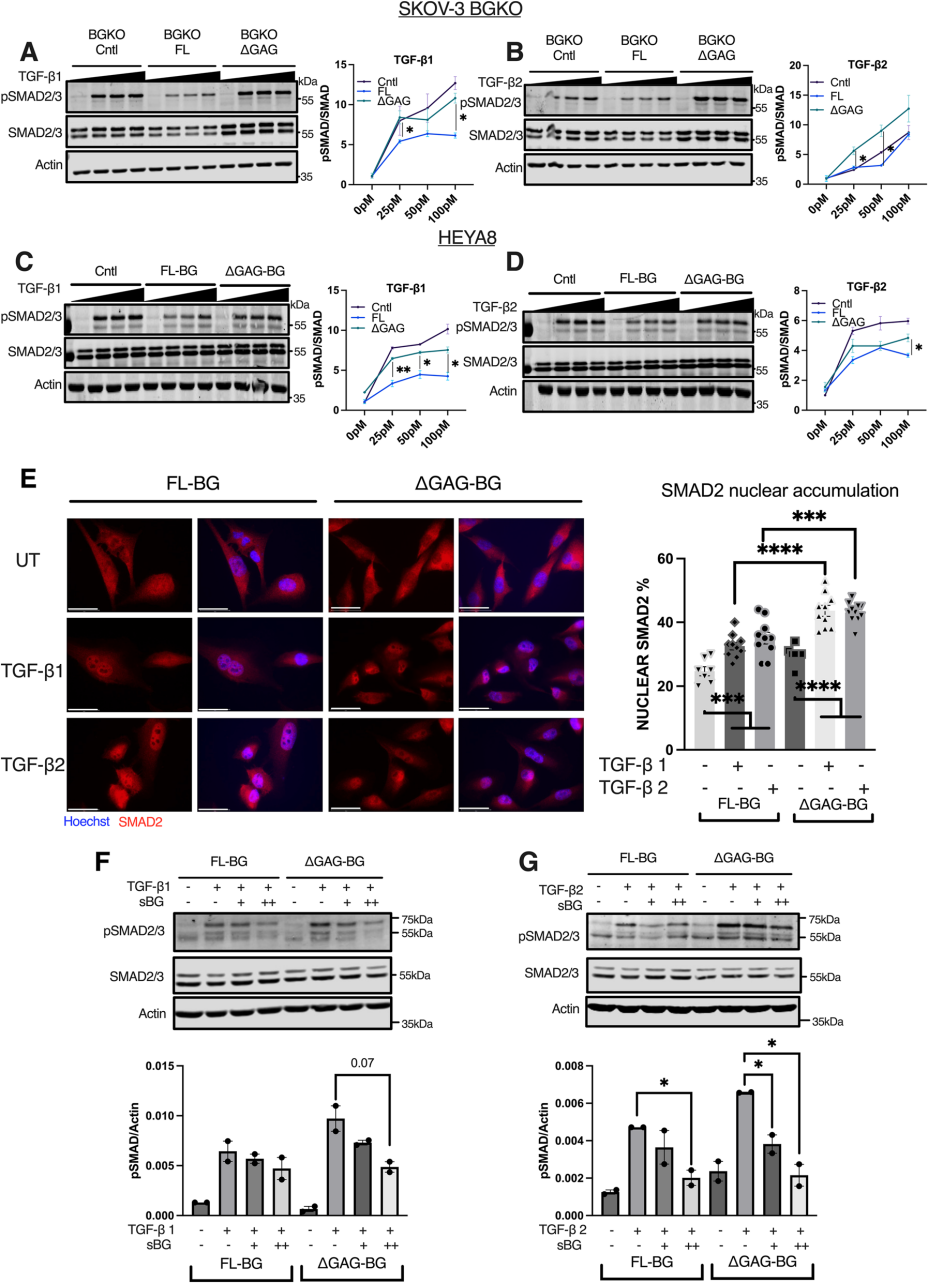
Suppression of TGF-β signaling by BG is dependent on the presence of its GAG chains. **A**, **B** Western blot and signal quantification of SMAD2/3 phosphorylation in SKOV-3 BGKO cells expressing either control vector (BGKO-Cntl), FL-BG (BGKO-FL), or ∆GAG-BG (BGKO-∆GAG) treated with increasing doses of **A** TGF-β1 or **B** TGF-β2 (25 pM to 100 pM). All signaling quantifications were performed by normalization of phospho-SMAD2/3 to the total SMAD2/3 signal. Phospho-SMAD signal normalized to SMAD signal is plotted by Mean ± SEM, (n = 3 combined trials). *p < 0.05, unpaired t-test between FL-BG and ∆GAG-BG within the same TGF-β treatment dose. (TGF-β1 at 25 pM, *p* = 0.0238, 100 pM, *p* = 0.0214, FL-BG vs. ∆GAG-BG. TGF-β2 at 25 M, *p* = 0.0167, 50 pM TGF-β2, *p* = 0.0185, FL-BG vs. ∆GAG-BG.) **C**, **D** Western blot and signal quantification of SMAD2/3 phosphorylation in HEYA8 cells expressing either control, FL-BG, or ∆GAG-BG, treated with increasing doses of **C** TGF-β1 or **D** TGF-β2 (25 pM to 100 pM). All signaling quantifications were performed by normalization of phospho-SMAD2/3 to the total SMAD2/3 signal. Phospho-SMAD signal normalized to SMAD signal is plotted by Mean ± SEM, (*n* = 3 combined trials). *p < 0.05, **p < 0.01, unpaired t-test between FL-BG and ∆GAG-BG within the same TGF-β treatment dose. (TGF-β1 at 25 pM, *p* = 0.0093, 50 pM, *p* = 0.0.193, 100 pM *p* = 0.0174, FL-BG vs. ∆GAG-BG. TGF-β2 at 100 pM, p = 0.0445, FL-BG vs. ∆GAG-BG.) **E** Representative immunofluorescence images of SMAD2 or nuclei (Hoechst) in response to 25 pM of TGF-β1 or TGF-β2, in HEYA8 cells. Scale Bar = 50 μm. Quantification of nuclear accumulation of SMAD2 was analyzed using cell profiler. The ratio of nuclear SMAD2 compared to total cellular SMAD2 is presented. Mean ± SEM, (n = 7 replicates). ****p < 0.0001, One-way ANOVA followed by unpaired t-test. (*p* < 0.0001, TGF-β1, and *p* = 0.0007, TGF-β2, FL-BG vs ∆GAG-BG.) **F**, **G** Western blot of phospho-SMAD2/3 in FL-BG and ∆GAG-BG expressing HEYA8 cells treated with 25 pM **F** TGF-β1 or **G** TGF-β2 either alone or in combination with ( +) 200 pg/mL to (+ +) 400 pg/mL of recombinant sol-BG. Phospho-SMAD2/3 signal normalized to actin signal is plotted by Mean ± SEM, (*n* = 2). *p < 0.05; **p < 0.05, unpaired t-test between TGF-β treated compared to TGF-β + sol-BG combination. (*p* = 0.0217, FL-BG TGF-β2 treated vs. TGF-β2 + 400 pg/mL sol-BG, and p = 0.0302, ∆GAG-BG TGF-β2 treated vs. TGF-β2 + 200 pg/mL sol-BG, *p* = 0.0171, ∆GAG-BG TGF-β2 treated vs. TGF-β2 + 400 pg/mL sol-BG)

Soluble/shed betaglycan has been shown to sequester TGF-βs’ [17, 39, 43]. Hence, we tested if ΔGAG-BG influences signaling and phenotypic TGF-β responses. Prior studies indicate maximum phosphorylation of SMAD2/3 by 30 min [64, 65] with OVCA cells showing similar kinetics of phosphorylation of SMAD2/3 at 30 min (Suppl. Figure 3A). Hence using this duration (30 min), we tested a dose range of TGF-β concentrations required to phosphorylate SMAD2/3. Expression of BG-FL in BGKO cells led to a 40 – 55% reduction in TGF-β1-induced phosphorylation of SMAD2/3, compared to control (BGKO-Cntl) cells (Fig. 3A). Similarly, compared to control HEYA8 cells, BG-FL expressing cells suppressed phosphorylation of SMAD2/3 in response to TGF-β1 treatment by 40% at the lowest dose of 25 pM and continued to suppress by 60% at higher doses of 100 pM as well (Fig. 3C). In response to TGF-β2, FL- BG suppressed SMAD2/3 phosphorylation significantly at both low and high doses depending on the cell line (Fig. 3B, D, Suppl. Figure 3E). Expression of ΔGAG-BG however showed a complete lack of the suppression of TGF-β1 signaling seen in FL-BG cells consistently across all cell lines (Fig. 3A, C, Suppl. Figure 3D). In the case of TGF-β2 as well, ΔGAG-BG did not suppress TGF-β2 induced SMAD2/3 signaling (Fig. 3B, D, Suppl. Figure 3E). Notably, ΔGAG-BG expressing cells in BGKO cells (BGKO-∆GAG), led to a further increase in TGF-β2 signaling even as compared to control cells (Fig. 3B). To determine if the SMAD2/3 phosphorylation responses mirrored TGF-β receptor activation, we tested the phosphorylation of type-I TGF-β receptor at Ser165 (pTGFBRI) upon treatment of the FL-BG and ∆GAG-BG expressing cells with increasing doses of TGF-β1 or TGF-β2 (Suppl. Figures 3G, H). Congruent with SMAD2/3 phosphorylation findings (Fig. 3C, D), FL-BG expressing cells exhibited suppression of TGFBR1 phosphorylation compared to control cells. Expression of ∆GAG-BG had similar activation of TGFBR1 upon TGF-β1 treatment compared to control cells. In the case of TGF-β2 treatment, 25 pM of TGF-β2 had greater activation of TGFBR1 in ∆GAG-BG expressing cells even compared to control or FL-BG cells (Suppl. Figure 3G, H). These data together suggest that the loss of GAG chains on BG led to a complete failure to suppress TGF-β1 signaling and an enhancement of TGF-β2 signaling at the level of receptor activation.

To examine if increased SMAD2/3 phosphorylation seen in ∆GAG-BG-expressing cells led to SMAD2/3 nuclear accumulation, we examined SMAD2 localization in stable HEYA8 cell lines expressing either FL-BG or ΔGAG-BG. SMAD2 alone was chosen for immunostaining compared to both SMAD2/3 as SMAD3 can accumulate in the nucleus regardless of the level of phosphorylation of receptors by TGF-βs. We used 25 pM of TGF-βs 1 and 2, the minimum dose required to detect differences in SMAD2/3 phosphorylation in (Figs. 3A-D) and allowed for 1 h of nuclear accumulation of SMAD2. A 1-h time point was chosen as prior studies indicate a minimum of 45 min for maximal SMAD2 retention in the nucleus [66, 67]. In the absence of exogenous ligands, FL-BG and ∆GAG-BG expressing cells showed 25% and 30% of total SMAD2 respectively in the population to be in the nucleus (Fig. 3E). In FL-BG-expressing cells, TGF-β1 or TGF-β2 treatment increased nuclear SMAD2 marginally, to 32% and 35% respectively. This contrasts with control vector-expressing cells that showed 35% nuclear SMAD2 accumulation at steady state (Suppl. Figure 3C) with both TGF-β1 and 2 treatments increasing the nuclear SMAD2 accumulation to 45% and 41% (Suppl. Figure 3C). However, in ∆GAG-BG cells, both TGF-β1 or TGF-β2 treatment increased nuclear SMAD2 to 43% which was significantly higher than FL-BG cells treated with TGF-β1 or TGF-β2 (Fig. 3E) and to a similar extent as control vector cells (Suppl. Figure 3C). This result reinforces the TGF-β1,2 signaling suppression role of FL-BG that was abrogated by ∆GAG-BG cells.

We next determined if shedding of BG within 30 min of TGF-β treatment was responsible for the reduction of TGF-β signaling in FL-BG expressing cells. We first assessed the amount of shed betaglycan in the conditioned media of HEYA8 Control Vector, FL-BG, and ∆GAG-BG stably expressing cells in a shorter, time frame of 1 min to 30 min. We find that by 30 mins at steady state, FL-BG media contained 170 pg/mL, whereas ΔGAG-BG expressing cells shed 50 pg/mL (Suppl. Figure 3F). Notably, 200 pg/mL of exogenous recombinant sol-BG (similar concentration of shed-BG at 30 min in FL-BG media) was sufficient to reduce TGF-β1,2 signaling in the ΔGAG-BG expressing cells (Fig. 3F, G). These data indicate that increased TGF-β1 and TGF-β2 signaling in ΔGAG-BG cells could be fully reduced to FL-BG levels by increasing the levels of soluble BG.

**Fig. 4:**
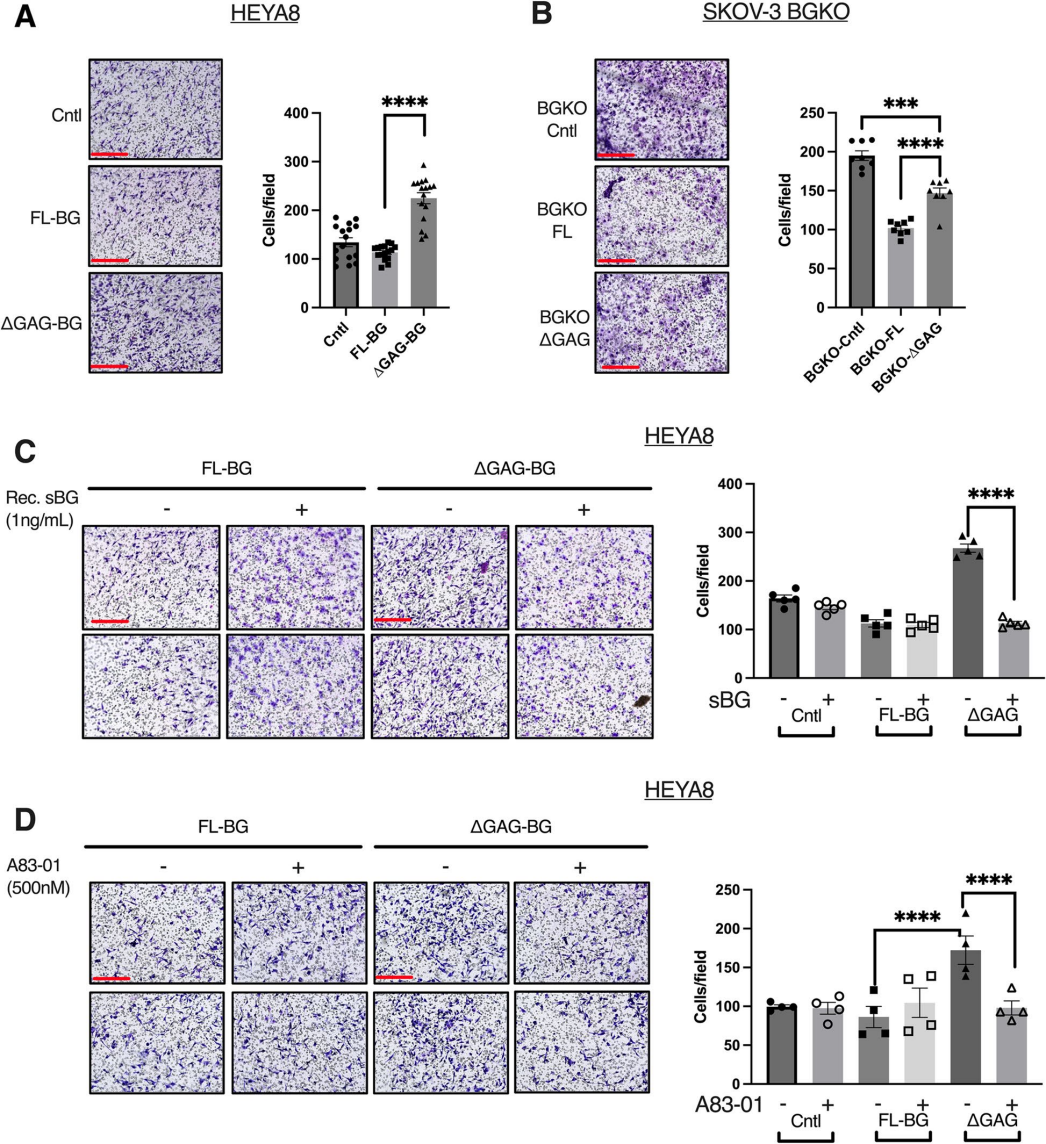
Unmodified BG (∆GAG) promotes invasion in a TGF-β signaling-dependent manner. **A** Representative images of invasion through Matrigel-coated Boyden-transwell inserts of HEYA8 cells expressing control vector, FL-BG or ∆GAG-BG, scale bar = 275 μm, and quantification of invaded cells. **B** Representative images of invasion through Matrigel-coated Boyden-transwell inserts of SKOV-3 BGKO cells as indicated and quantification of invaded BGKO cells. Mean ± SEM are plotted, (HEYA8 Stable n = 16, SKOV-3 BGKO, BGKO-FL, and BGKO-∆GAG, *n* = 8) ***p < 0.001; ****p < 0.0001, One-way ANOVA followed by unpaired t-test between FL-BG and ∆GAG-BG. (*p* < 0.0001, HEYA8 Stable FL-BG vs ∆GAG-BG, *p* < 0.0001, SKOV-3 BGKO-FL vs. BGKO-∆GAG). (**C**) Representative images of invasion through Matrigel-coated Boyden-transwell inserts of HEYA8 FL-BG and ∆GAG-BG cells treated with soluble recombinant BG at 1 ng/mL in the top chamber. Scale Bar = 275 μm and quantification of the invaded. Mean ± SEM are plotted, (*n* = 5), ****p < 0.0001, One-way ANOVA followed by unpaired t-test between sBG untreated to sBG treated groups. (*p* < 0.0001 ∆GAG-BG untreated vs. ∆GAG-BG, sBG treated). (**D**) Representative images of invasion through Matrigel-coated Boyden-transwell inserts of HEYA8 FL-BG and ∆GAG-BG cells treated with 500 nM of A83-01 in the top chamber and quantification of invaded cells. Mean ± SEM were plotted, (*n* = 4), ****p < 0.0001, One-way ANOVA followed by unpaired t-test between untreated compared to A83-01 treated groups. (*p* < 0.0001 ∆GAG-BG untreated vs. ∆GAG-BG, A83-01 treated)

BG has been in several prior studies shown to be a strong regulator of both TGF-β dependent and independent cell migration and invasion responses [13, 49, 68]. Since ΔGAG-BG sheds less and does not suppress TGF-β signaling, we tested the impact of FL-BG and ∆GAG-BG expression on cellular motility and invasion of ovarian cancer cells and the effect of TGF-β signaling on the same. We find that FL-BG lowered invasion in HEYA8 cells (Fig. 4A) that reached significance in CRISPR SKOV-3 BGKO cells which had FL-BG expression restored (BGKO-FL, Fig. 4B). In contrast, ΔGAG-BG expression in both cell lines (HEYA8 and SKOV-3 BGKO cells) showed an increase in cellular invasion as compared to FL-BG mutants (2X  in HEYA8 stable and transient, and a 1.4X increase in SKOV-3 BGKO, as well as in SKOV-3 Stable BG expressing cells, Fig. 4A, B, Suppl. 4A, B). Next, we sought to test whether exogenous shed-BG impacts cellular motility and invasion in FL-BG and ∆GAG-BG-expressing cells. We find that exogenous sBG suppressed invasion of ΔGAG expressing cells by 60%, compared to untreated ΔGAG cells (Fig. 4C), whereas FL-BG expressing cells were not affected by exogenous shed-BG, indicating that the increased invasion in ΔGAG-BG expressing cells was, in part, due to reduced shedding of BG in ∆GAG-BG expressing cells. Moreover, to identify, whether the increased invasiveness of ∆GAG-BG expressing cells compared to FL-BG expressing cells is due to increased TGF-β signaling, we inhibited TGF-β signaling using A83-01, a small molecular inhibitor of ALK4,5,7 [69] (Suppl. Figure 4C) and tested for cellular invasion. A83-01 treatment led to a 50% reduction in invasion of ∆GAG-BG cells compared to untreated cells (Fig. 4D). No significant effect of the inhibitor was seen in either the control vector or FL-BG-expressing cells (Fig. 4D). These data indicate that the reduced shedding and increased TGF-β signaling in the BG-∆GAG cells is a direct contributor to increased invasion in cells that express unmodified ∆GAG-BG.

### GAG-dependent BG ectodomain shedding is negatively regulated by TIMP3

We sought to delineate a mechanism for enhanced HS modification-dependent BG ectodomain shedding. Since shedding differences were seen under steady-state growth conditions, genome-wide gene expression profiles of HEYA8 control-vector cells as compared to ΔGAG, S534A-CS BG, and S545A-HS BG were compared using transcriptomics from cells under steady-state and regular growth media conditions. Single-chain mutants were tested instead of FL-BG mutants to differentiate chain-specific responses rather than a heterogeneous mixture of CS, HS, and ΔGAG that are present in FL-BG-expressing cells. We focused on differentially expressed genes that belonged to either MMP or ECM-related genes to identify potential regulators of shedding. Our analysis revealed only a small subset of genes (*n* = 9: *ADAMTS17*, *TMPRSS11CP*, *MMP3*, *TIMP3*, *PRSS48*, *PRSS36*, *ADAM8*, *ADAMTS3*, *MMP23B*) that were differentially expressed when comparing HS-BG and CS-BG against ∆GAG-BG expressing cells that belonged to either MMP or ECM-related mechanisms (Suppl. Table 1).

**Fig. 5:**
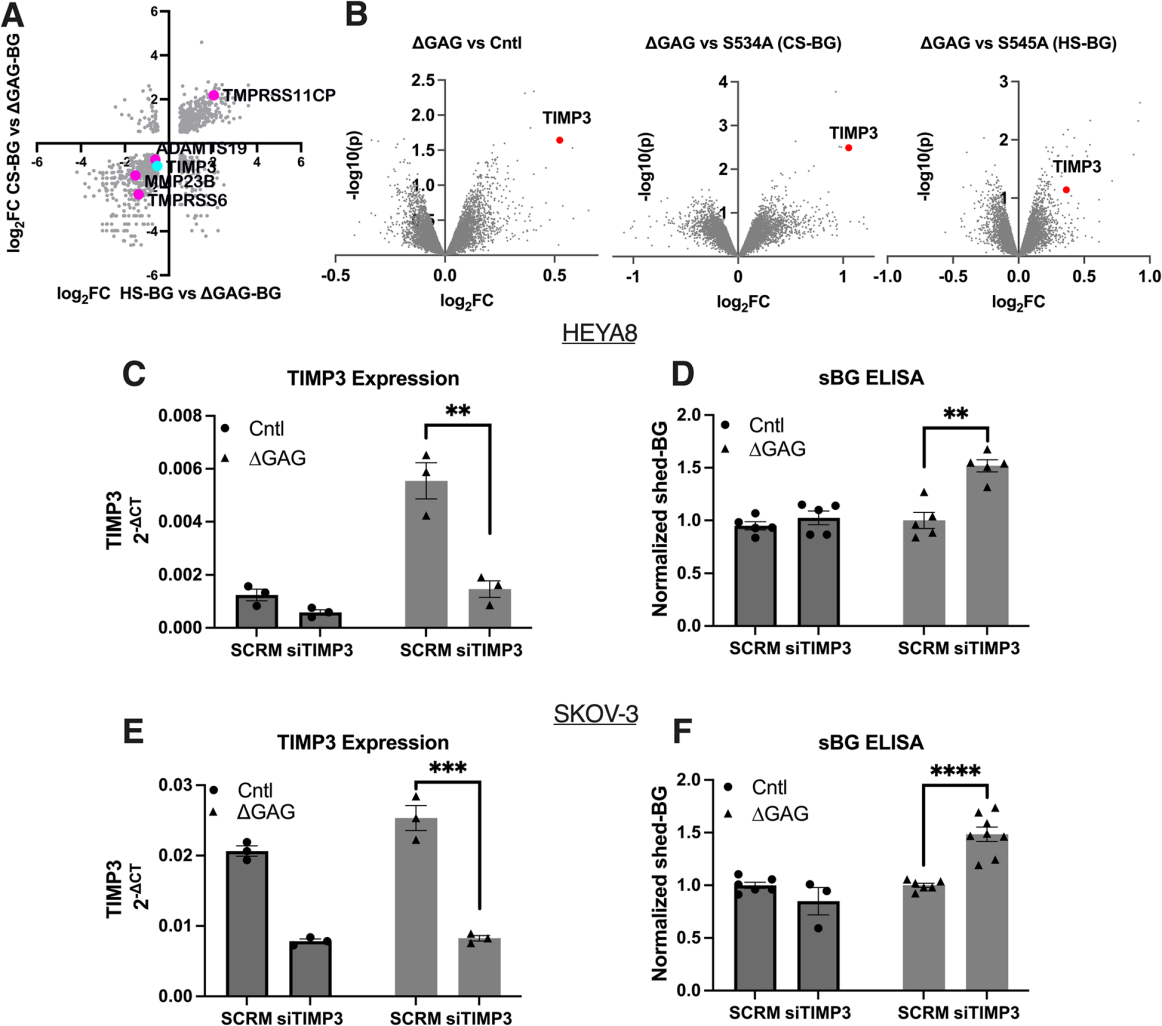
TIMP3 negatively regulates glycosaminoglycan-dependent BG ectodomain shedding. **A** Scatter plot of the differentially expressed genes from transcriptomics of HS-BG and ∆GAG-BG plotted against CS-BG and ∆GAG-BG comparison groups. All protease-related genes are highlighted. **B** Volcano plots of the differentially expressed genes between ∆GAG-BG vs. Control-vector, S534A (CS-BG) vs. ∆GAG-BG, and S545A (HS-BG) vs. ∆GAG-BG. (*TIMP3*; ∆GAG vs Cntl, log_2_FC = 0.524, -log(10)*p* = 1.643, ∆GAG vs CS-BG, log_2_FC = 1.054, -log(10)*p* = 2.493, ∆GAG vs HS-BG, log_2_FC = 0.364, -log(10)*p* = 1.1411.). **C**, **E** Semi-qRT-PCR of *TIMP3* and (**D**, **F**) ELISA for sol-BG in indicated cells normalized to the scramble vector  cells. Mean ± SEM (*n* = 3 qRT-PCR), *p < 0.05; **p < 0.01; ***p < 0.001; ****p < 0.0001, One-way ANOVA followed by unpaired t-test between scramble vector treated cells to siTIMP3 treated cells. (HEYA8 *p* = 0.0055 ∆GAG-BG SCRM vs siTIMP3, SKOV-3 *p* = 0.0007, ∆GAG-BG SCRM compared to siTIMP3), for ELISA in (D, F) *n* = 5 HEYA8, *n* = 6 SKOV-3, *p* = 0.0068, ∆GAG-BG SCRM vs siTIMP3 in HEYA8 and *p* =  < 0.0001, ∆GAG SCRM vs. siTIMP3 in SKOV-3

Most notably, within the small subset of altered genes at steady state, tissue inhibitor of metalloproteinase-3 (*TIMP3*) was found to be most expressed in ΔGAG-BG as compared to both S534A CS or S545A HS-BG mutant expressing cells (p < 0.05.) (Fig. 5A). Interestingly, we also noted that BG/*TGFBR3* levels were significantly higher in modified (single chain) expressing cells as compared to ∆GAG-expressing cells in the transcriptomics (Suppl. Table 1), however, this increase in *TGFBR3* mRNA was not observed at the protein level as determined by [^125^ I]-TGF-β1 binding and crosslinking (Fig. 2C). We focused on TIMP3 changes and used semi-quantitative qRT-PCR and western blotting to extend the findings of the transcriptomics on TIMP3 levels to additional cell lines and in cells expressing either ΔGAG-BG or FL BG. We find that *TIMP3*-RNA was significantly higher in ΔGAG-BG cells as compared to FL BG cells in both HEYA8 and SKOV-3 cells (Suppl. Figure 5A, B).

TIMP3 protein levels were also higher in SKOV-3 ΔGAG-BG (Suppl. Figure 5C) but were not detectable in HEYA8 cells. Next, to test a direct role for TIMP3 and its inhibitory effects on BG ectodomain shedding, we utilized siRNAs to target TIMP3 in two cell lines (HEYA8 and SKOV-3) expressing control vector, FL-BG, and ΔGAG-BG. siRNAs to TIMP3 compared to scramble vector led to lowered TIMP3 (70–80% reduction) expression (Fig. 5C, E). BG ELISA of conditioned media from scramble vector cells or siTIMP3 cells revealed a 51% and 28% increase in shed-BG from the ∆GAG-BG expressing HEYA8 and SKOV-3 cells respectively upon knockdown of TIMP3 (Fig. 5D, F). Our findings thus demonstrate TIMP3 as a regulator of BG ectodomain shedding, that is dependent on the GAG chain-mediated steady-state differences in TIMP3 levels.

### Shed BG is found in patient ascites fluid and correlates with patient outcomes and TGF-β responses

To assess the clinical significance of shed-BG we assessed the levels and type of BG (if any) in the ascites fluid of ovarian cancer patients. For this, acellularized ascites fluid (AF) of advanced OVCA patients banked at three different institutions was assessed by ELISA for soluble BG to account for any differences associated with the collection and banking of samples (Duke, Penn State, and UAB). A total of 60 samples were tested. Individual patient AF information as well as staging, survival, and histology information can be found in Suppl. Table 2.

**Fig. 6:**
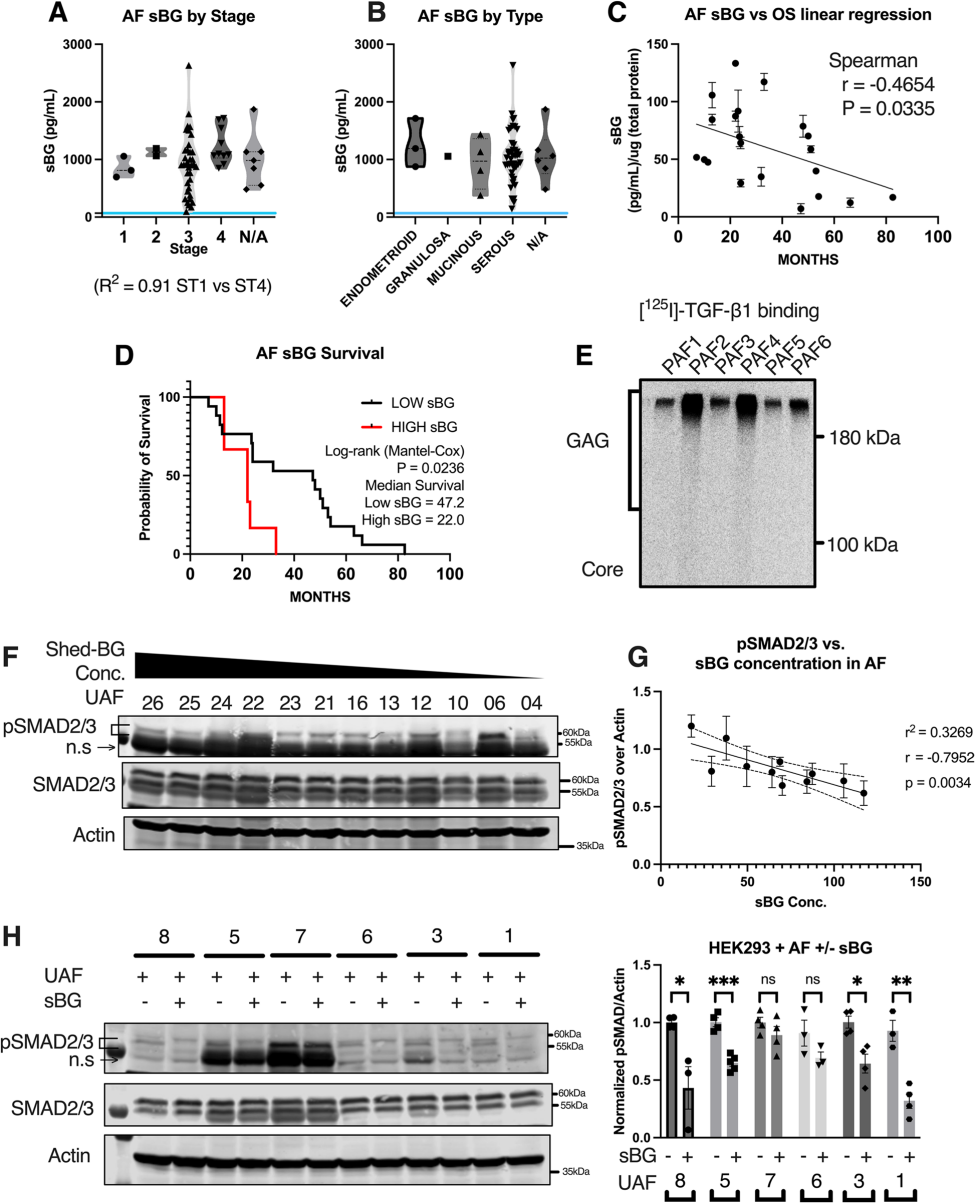
Glycosaminoglycan-modified soluble–betaglycan (sBG) in ascites fluid is associated with decreased survival and TGF-β signaling responses for ovarian cancer patients. **A**, **B** Sol-BG concentration in AF samples plotted by **A** tumor stage, and **B** tumor type. Each data point represents a single patient sample. The blue line indicates the average sol-BG concentration in the plasma of healthy volunteers. **C** Correlation analysis of sol-BG concentration in the ascites fluid compared to patient overall survival in months. (*n* = 21) Spearman correlation analysis was performed, r = -0.4654, *p < 0.05; (*p* = 0.0335). **D** Kaplan–Meier survival plot of OVCA patients stratified into low (< 30th percentile) and high sol-BG (> 70th percentile) groups. A log-rank (Mantel-Cox) test was performed, *p* = 0.0236, median survival Low sol-BG = 47.2 months, High sol-BG = 22.0 months. **E** Autoradiograph of patient ascites fluid following [^125^ I]-TGF-β1 binding and crosslinking and immunoprecipitation using anti-BG antibody. **F** Western blot for SMAD2/3 phosphorylation in HEK293 cells treated with indicated OVCA patient ascites fluid. ns: nonspecific band **G** Correlation graph of SMAD2/3 phosphorylation in HEK293 cells, to the sol-BG concentration in the AF, used to treat HEK293 cells. Pearson correlation and simple linear regression were performed. (Pearson *r* = -0.7952, r^2^ = 0.3269, *p* = 0.0034). **H** Western blot of SMAD2/3 phosphorylation in HEK293 cells treated with OVCA patient ascites fluid with low sol-BG concentrations (< 400 pg/mL), with/without 1 ng/mL recombinant sol-BG and quantification of phospho-SMAD2/3 from (H) normalized to untreated samples and actin is plotted by Mean ± SEM, (*n* = 3), unpaired t-test between recombinant sBG untreated to treated samples. ns: nonspecific band *p < 0.05; **p < 0.01; ***p < 0.001; ****p < 0.0001. One-way ANOVA followed by unpaired t-test between sBG untreated (-) to treated ( +). (*p* = 0.0152 UAF8, *p* = 0.0002 UAF5, *p* = 0.2577 UAF7, *p* = 0.1576 UAF6, *p* = 0.0101 UAF3, *p* = 0.0027 UAF1)

The range of sol-BG was found to be between 96 pg/mL to 2600 pg/mL (Suppl. Figure 6A). The average concentration of sol-BG across all repositories was 993 pg/mL, with a median of 915 pg/mL (Suppl. Figure 6A), suggesting that potential differences in banking the fluid and age of fluid did not impact the detection of sol-BG. We thus combined the data and found that Stage 1 patients contained a mean of 854 ± 184 pg/mL sBG, and Stage 2 patients had a mean of 1128 ± 90 pg/mL. The highest variability was seen in stage 3 patients, with a mean of 980 ± 530 pg/mL, and a range from 95 pg/mL at the lowest to 2632 pg/mL at the highest. Stage 4 patient samples showed the highest mean concentration of 1221 pg/mL (± 463 pg/mL). In comparison, serum from healthy volunteers (*n* = 14) showed a range of 34-100 pg/mL with a median of 66.7 pg/mL (Fig. 6A**,** blue line). Although patient numbers in each stage were variable, the amount of sol-BG positively correlated with the disease stage (R^2^ = 0.91 ST1 vs ST4) (Fig. 6A), indicating accumulation of sol-BG with increasing disease stage. We also categorized the samples by disease type but did not find a correlation between the type of disease and the sol-BG amount (Fig. 6B). However, sol-BG levels negatively correlated (*r* = -0.4654) with patient survival (in months) (Fig. 6C). Stratifying the patients into low or high BG groups followed by survival analysis revealed that ‘High sol-BG’ patients had the lowest median survival (22 months) compared to ‘Low sol-BG’ patients (47.2 months) (Fig. 6D). Since stage 3 patients account for the majority of the patient samples, we also performed a survival analysis exclusively of the stage 3 patients (Suppl. Figure 6B). We find that median survival months for ‘High sol-BG’ patients remained at 22 months, and was 47.2 months for ‘Low sol-BG’ patients.

We next assessed the modification status of BG in the patient ascites fluid. We find ascites-derived BG to be glycosaminoglycan modified with effective TGF-β1 binding (Fig. 6E). Ascites-derived sol-BG presented with both HS and CS GAG chains (Suppl. Figure 6C) similar to when expressed in vitro in cell lines (Fig. 1A**, **Suppl. Figure 2), as determined by Heparinase and Chondroitinase enzymatic digestion to quantify the percent distribution of GAG chains of sol-BG in the ascites fluid (Suppl. Figure 6C). To assess if sol- BG in the fluid impacted TGF-β ligand availability for cell signaling, HEK293 cells were treated with patient fluid (*n* = 12) for 30 min and analyzed for phosphorylation of SMAD2/3 (Fig. 6F). AF samples were chosen based on their sBG levels ranging from the highest sBG concentration at 2600 pg/mL to the lowest at 250 pg/mL (Suppl. Figure 6D, Suppl. Table 2). We find that cells treated with UAF26, which contains the highest amount of sBG at 2600 pg/ml, had the least amount of SMAD2/3 phosphorylation, compared to cells treated with UAF06, containing sol-BG at 316 pg/mL which showed the highest SMAD2/3 phosphorylation. However, not all samples impacted SMAD2/3 phosphorylation based on the sol-BG levels as UAF04, (sBG concentration at 250 pg/mL), did not lead to SMAD2/3 phosphorylation in HEK293 cells. A correlation analysis of the SMAD2/3 signal in HEK293 cells compared to the concentration of the sol-BG in the ascites fluid that was used to treat HEK293s showed a negative correlation between sol-BG in the patient AF and SMAD2/3 phosphorylation in HEK293 cells (*r* = -0.7952) (Fig. 6G). To conversely test if SMAD2/3 phosphorylation in 293 cells in response to AF could be suppressed by the addition of recombinant sol- BG, we added 1 ng/mL (average of sBG concentration within the patient AF samples (Fig. 6H), to the AF with the lowest sol-BG levels (Suppl. Figure 6E, Suppl. Table 2). Combination of AF with 1 ng/mL rec. sol-BG diminished the ability of the AF to stimulate phosphorylation of SMAD2/3 by an average of 40% in 293 cells (Fig. 6H). These data indicate that sol-BG in advanced OVCA patient ascites fluid is glycosaminoglycan modified and negatively correlates with patient survival. Notably, AF samples with low sBG can activate TGF-β signaling in a paracrine manner more effectively than AF from patients with high sBG. These data together suggest that sol-BG could serve as a measure of TGF-β responsiveness in patients.

## Discussion

Here, we investigated the effects of glycosaminoglycan chains, either heparan sulfate or chondroitin sulfate modified, on BG ectodomain shedding and TGF-β signaling and discovered that heparan sulfate modified GAG chains are crucial for betaglycan ectodomain shedding. We also found that at baseline, TIMP3 expression is higher in cells expressing BG without the GAG chains, which we demonstrate to be a negative regulator of betaglycan shedding, thereby affecting TGF-β1/2 signaling responses. Importantly, we have unequivocally demonstrated for the first time a critical link between post-translational glycosaminoglycan modifications of betaglycan and TGF-β signaling responses. Our study highlights the significance of our findings to ovarian cancer, as modified betaglycan is present in the ascites fluid of ovarian cancer patients and can be used for predicting patient survival and TGF-β signaling responses. These findings are also likely to have much broader implications for additional tissues and pathologies such as those involving the endometrium, where BG shedding is tightly regulated by ligands [70, 71] in the prostate where it influences osteogenic programs [16], and even in tissue renewal mechanisms where BG was recently identified as a unique marker for self-renewing muscle satellite cells [72].

One of the earliest studies on soluble BG by Fukushima et al. [73] found that a fragment of the transmembrane domain (AA 788–769) of betaglycan containing the TGF-β binding region, increased TGF-β binding to the type-II receptor when administered as a soluble protein. Although the study demonstrated a TGF-β signaling enhancing role for soluble betaglycan, this effect was only visible at low concentrations, with high concentration of fragment-sol BG leading to sequesteration of TGF-β from the type II and type I receptors. Since then, several studies in other models demonstrated that sol-BG blocks TGF-β signaling. Additionally, sol-BG studies in disease models reinforces the inhibitory role of sol-BG on TGF-β signaling mediated invasion and migration [19, 28, 71, 74, 75], as well as downregulation of TGF-β responsive genes [76]. It is possible that small amounts of shedding versus higer degree of shedding may have similar dichotomous effects on signaling that needs to be determined in diease models in the future.

The observation that GAG chains are required for maximum BG ectodomain shedding across ovarian cancer cell lines, as well as in noncancer cells (Figs. 1 and 2) suggests that this may not be a tumor cell-specific phenomenon and could occur in host tissues in the tumor environment. We also observed that the GAG-modified BG in OVCA presents predominantly with heparan sulfate chains which also appears to be a stronger driver of shedding as compared to the CS modification (Fig. 2E). This finding was confirmed using two independent approaches that included site-directed mutagenesis of the specific GAG attachment/modification sites of BG at ser-534 and ser-545 (Fig. 2E) and utilization of CHO-K1 and its mutant cell lines devoid of HS-GAG synthesis (CHO-677, mimicking CS-BG or CHO-745, mimicking ∆GAG-BG) (Figs. 2A, B). However, the impact of the extent of the sulfation, sulfation patterns of the glycosaminoglycan chains, and the alteration of the glycosaminoglycan chain structure on BG shedding and signaling have not been investigated and remain to be determined. It is unclear if the CS-modified GAG chains have alternate roles, such as mediating association with ECM components as has been reported for other PGs such as NG2 [77]. Other cell surface heparan sulfate proteoglycans (HSPG), such as syndecans, perlecans, and glypicans have been shown to promote cancer pathogenesis (including ovarian) and expression changes of such HSPGs have been correlated with poorer patient outcomes [78–80]. Additionally, aberrant regulation of heparan sulfate sulfotransferases, responsible for enzymatic modification of HS on all HS proteoglycans, has been known to affect several pathophysiological processes from inflammation to organ development and cancer [45, 81]. suggesting that shifting the balance to a more HS-modified BG could be common in pathologies. Sulfation patterns on proteoglycans  [45, 48, 82] can influence the accessibility of proteolytic enzymes, and alter the interactions with ligands [83].

Recent studies have shown differential effects of BG’s GAG chains in FGF2 and Wnt3A signaling. Sulfation of the GAG chains on BG was shown to impact the effect of BG on Wnt3a signaling, wherein non-sulfated GAG chains of BG stimulated Wnt3a signaling [44]. CS-modified BG stimulated the accumulation of β-catenin, increasing TCF/LEF transcription activity whereas HS-modified BG had an opposing effect by suppressing Wnt3a signaling [44]. A potential BG-Wnt5a axis has also been reported [16] and the effects of GAG chains and sulfation for Wnt5a remain unexamined. BG also binds FGF2 through GAG chains [50, 54]. In neuroblastoma models, FGF-2 mediated differentiation was dependent on HS-modified BG, specifically 2-O sulfated glucuronic and N-sulfated glucosamine [50] and complex formation with FGF receptors and BG was GAG chain-dependent [54, 50]. Highly sulfated regions of HS, also known as s-domains [84], have been shown to bind FGF. In addition, HS chains could also serve as a scaffolding molecule [85]. Our study does not fully investigate all known and possible binding partners and other growth factors with affinities for GAGs. The effect of GAG modifications are thus likely to influence signaling not just of TGF-β, but also Wnts and FGF2, leading to potential cross talk mechanisms that could be fine tuned by betaglycan particularly in the case of HS modifications and sulfation patterns that we previously reported to regulate FGF2 and Wnt3A signaling.

Previous reports have shown that the affinity of TGF-β1 to BG devoid of GAG chains are comparable to those of wild-type BG [39] leading to the long-standing view that the GAG modifications do not significantly contribute to TGF-β signaling responses. However, our findings challenge this view , as we find that GAG chains influence shedding which has an undeniable effect on the sequestration of ligands by shed-BG, thereby influencing signaling (Fig. 3F, G). Indeed, we anticipate that small changes in the amount of soluble betaglycan can contribute to large changes in TGF-β signaling. It is well established that the amount and duration of TGF-β signaling are both critical to responses [86–88]. A small increase in steady-state SMAD2 nuclear accumulation was seen in untreated ∆GAG-BG-expressing cells compared to untreated FL-BG-expressing cells (Fig. 3E). Congruently, we observed increased phopho-SMAD2/3 in ∆GAG-BG cells compared to FL-BG cells at a steady state in serum-containing media (Suppl. Figure 3B). Increased phosphorylation and increased nuclear accumulation of SMAD2 upon TGF-β 1&2 treatment in ∆GAG-BG cells compared to FL-BG cells support the direct influence on durable cell signaling. Our studies focused on SMAD2/3 signaling as a primary readout of TGF-βs 1/2. However, TGF-β s can elicit signaling through other transducers, such as SMAD1/5, and crosstalk with other signaling pathways via SMAD-independent pathways such as, but not limited to, ERK, JNK, P38, PI3K, Wnt, and Hh signaling pathways [89]. The impact of shed-BG and TGF-β superfamily growth factors, as well as crosstalk with other signaling pathways in OVCA and other diseases, remains to be determined.

Early studies analyzed the affinities of TGF-βs to membrane-bound BG and sol-BG and found similar affinities for TGF-β2 or 1 [9]. Binding of TGF-β to sBG or membrane-bound BG is likely to be competitive, however, the outcomes on signaling have been shown to be opposing. A prior study where BG shedding was abrogated and compared to a super shedder of betaglycan revealed that the non-shedder mutant of BG increased TGF-β induced SMAD signaling compared to a super shedder mutant of BG [19]. This is phenocopied by our findings when comparing the effects of ∆GAG-BG to FL- BG consistent with the hypothesis that membrane-bound betaglycan may increase the presentation of TGF-β ligands to the signaling receptors, whereas soluble betaglycan likely primarily sequesters ligands to inhibit signaling. A signaling kinetic study by Lopez-Casillas et al. demonstrated that soluble BG binds to TGF-βs and inhibits TGF-β binding to the membrane receptors. The addition of soluble betaglycan in combination with TGF-βs 1 or 2 inhibited binding to the signaling receptors [39]. We find that increasing the amount of soluble BG suppressed TGF-β 1/2 signaling in ∆GAG-BG cells (less shed BG) in a dose-dependent manner (Fig. 3F, G). The amounts of soluble BG chosen were based on quantitative assessments of the amount of BG shed within the time course of the studies (Suppl. Figure 3F). Our data are consistent with prior studies suggesting a competition between sol BG and membrane BG for TGF-β.

Our studies do not rule out the alternate possibility of differences in the ability of the fully modified (FL-BG) and unmodified (∆GAG-BG) BG to influence the stability of the cell surface receptor complexes formed between BG and TβRII, TβRI receptors for TGF-β signaling. This model was supported by a prior study [43], which indicated that the GAG chains of BG reduced the formation of TGF-β receptor complexes, thereby inhibiting signal transduction. Although we did not thoroughly investigate the formation of TGF-β receptor complexes between BG GAG mutants, affinity binding of [^125^ I]-TGF-β1 in BG FL and ∆GAG mutant expressing cells showed the presence of TGF-βRI and TGF-βRII when immunoprecipitated using an anti-BG antibody, suggesting that GAG-modified and unmodified BG interact with TGF-βRI and TGF-βRII. We showed a greater impact on the inhibition of TGF-β signaling by the ligand-sequestration effect of shed-BG (Figs. 3F, 3G). Based on our findings, a more quantitative assessment of the effect of the modifications on BG on receptor homo- heterodimerization is also warranted.

We and others published several prior studies on the effect of FL-BG on reducing tumor cell motility [5, 17, 24–26, 49] primarily via Cdc42, and interactions with β-arrestin2 [49, 58], and the potential influence of GAG chains [50, 54]. The expression of modified BG leading to inhibition of migration and the increased migration upon expressing unmodified BG was consistent with previous reports particularly in cells with BG knockout by CRISPR (BGKO) that had restored FL-BG (BGKO-FL) expression (Fig. 4B) suggesting that even small changes in the balance between modified and unmodified BG can shift the effects on tumor cell motility.

Considering the impact of the shift in the balance of shed versus unshed BG (modified and unmodified), it is worth noting that TIMP3, that we defined here as a regulator BG ectodomain shedding, is also altered in several cancers and has been proposed as a therapeutic target [90]. TIMP3 can inhibit a broad range of MMPs including MT-MMP 1–3 and based on prior studies on the effects of MT-MMPs 1–3 on the shedding of BG [29, 33, 75], we anticipate that the effects of MMPs’ are likely to be dependent on the levels of TIMP3. However, how TIMP3 is regulated in ∆GAG cells remains to be determined and could be downstream of the different ligands that are impacted either by the glycosaminoglycan chains on BG or the BG core binding ligands (Wnt and FGF or TGF-β superfamily respectively).

Under normal physiological conditions, BG can be detected in plasma [91] and milk [92], and has been shown to be a neutralizing agent for TGF-β. In the context of ovarian cancer, ascitic fluid (AF) accumulation in the peritoneum enables the transcoelomic tumor spread of metastatic cells [93]. Previous studies have shown the prognostic value of identifying components in AF to predict treatment and survival outcomes [94, 95]. AF from tumor-bearing mice and patients are known to have elevated TGF-β1 [96–98]. Our observation of the elevated amounts of soluble BG being associated with worse patient outcomes in conjunction with prior studies demonstrating lower BG expression in tumors suggests that there may be additional sources of soluble BG in the ascites. We propose that sol-BG in ascites, which can dull TGF-β signaling responses (Fig. 6F), could potentially serve as a predictor for patient response to TGF-β ligand-targeted therapies.

## Conclusion

In conclusion, heparan sulfate glycosaminoglycan modifications on betaglycan are critical for its ectodomain shedding, a feature indispensable for the suppression of TGF-β signaling and the cells' responses to exogenous TGF-β. We identified TIMP3 as a key negative regulator of betaglycan shedding and thereby TGF-β signaling. Modified betaglycan is present in soluble form in the ascites fluid of patients with ovarian cancer and is a marker of patient outcomes and TGF-β signaling responses. We demonstrate a novel reliance on the glycosaminoglycan chains of betaglycan for shedding and TGF-β signaling responses, crucial for understanding TGF-β signaling in cancer.

### Limitations of the study

The impact of GAG modifications of BG on TGF-β signaling was limited to the readout of phosphorylation of SMAD2/3 and not any non-SMAD dependent pathways which could be indirectly impacted by soluble betaglycan. Additionally, our study does not rule out the possibility of glycosaminoglycan chains of BG influencing any of the several GAG binding growth factors or the stability of the cell surface receptor complexes formed between BG and TβRII, and TβRI kinase receptors for TGF-β signaling. We also do not conduct in-depth structural analysis of HS chains on shedding. Lastly, although we show that patient ascites fluid contains ligands that can activate SMADs and the addition of recombinant shed-BG can suppress the SMAD2/3 phosphorylation, the composition, and precise ligands that activate SMADs have not been investigated here.

### Supplementary Information

**Additional file 1:** Supplementary Figure 1

**Additional file 2:** Supplementary Figure 2

**Additional file 3:** Supplementary Figure 3

**Additional file 4:** Supplementary Figure 4

**Additional file 5:** **Supplementary Table 1.** Differentially expressed MMP or ECM-related genes from RNA-SEQ

**Additional file 6:** Supplementary Figure 5

**Additional file 7:** **Supplementary Table 2.** Patient ascites fluid sample list

**Additional file 8:** Supplementary Figure 6

## Data Availability

All the sequencing data are publicly available and have been deposited at the Gene Expression Omnibus (GEO) accession number GSE237403. Original Western blot and microscopy images are available from the corresponding author upon request. This study does not report any original code. Any additional information and data for reanalysis is available from the corresponding author upon request.

## Abbreviations

TβRIII: Type III TGF-β receptor
BG: Betaglycan
TGF-β: Transforming Growth Factor Beta
GAG: Glycosaminoglycan
HS: Heparan sulfate
CS: Chondroitin sulfate
OVCA: Ovarian cancer
BG-FL: Full-length betaglycan
BG-∆GAG: Betaglycan lacking glycosaminoglycan chains
BGKO: CRISPR knockout of betaglycan
sBG: Shed betaglycan
sol-BG: Soluble betaglycan
CM: Conditioned media
qRT-PCR: Semi-quantitative RT-PCR
