# Supplementary figures and images for: Glycosaminoglycan modifications of betaglycan regulate ectodomain shedding to fine-tune TGF-β signaling responses in ovarian cancer
Source: Cell Commun Signal. 2024 Feb 15;22:128. doi: 10.1186/s12964-024-01496-y (PMC10870443; doi:10.1186/s12964-024-01496-y)

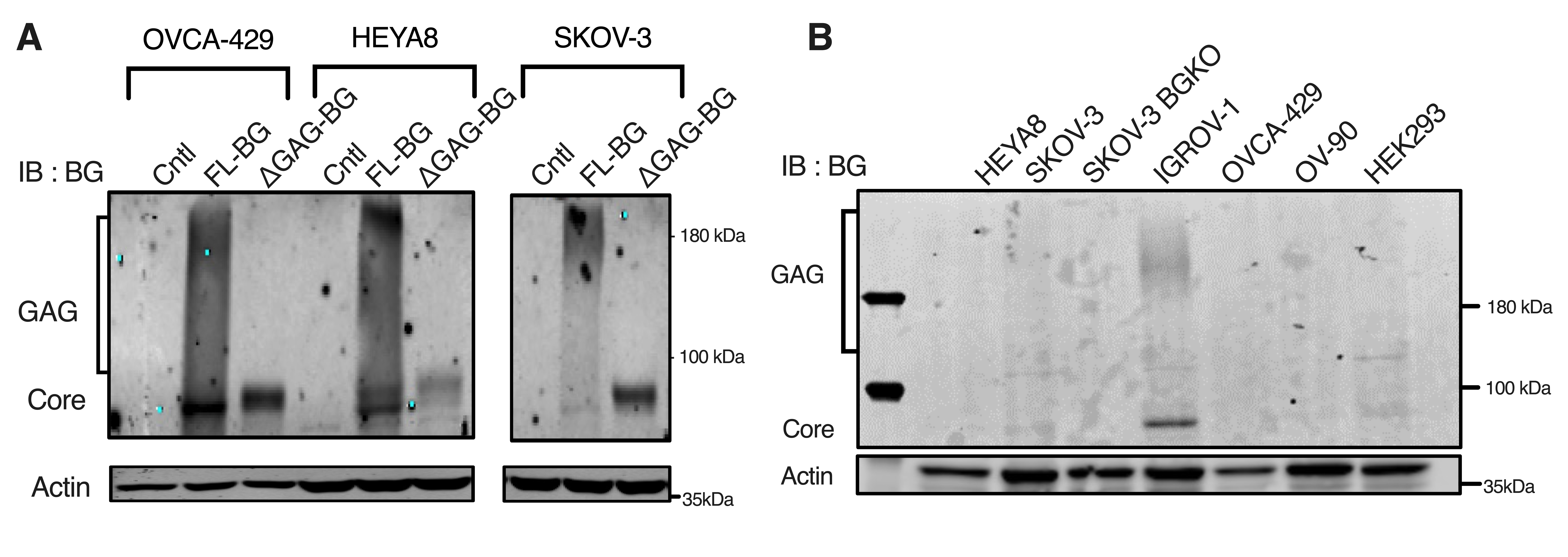

Supplement: Supplementary file 1 — Additional file 1: Supplementary Figure 1 [file 12964_2024_1496_MOESM1_ESM.tiff]

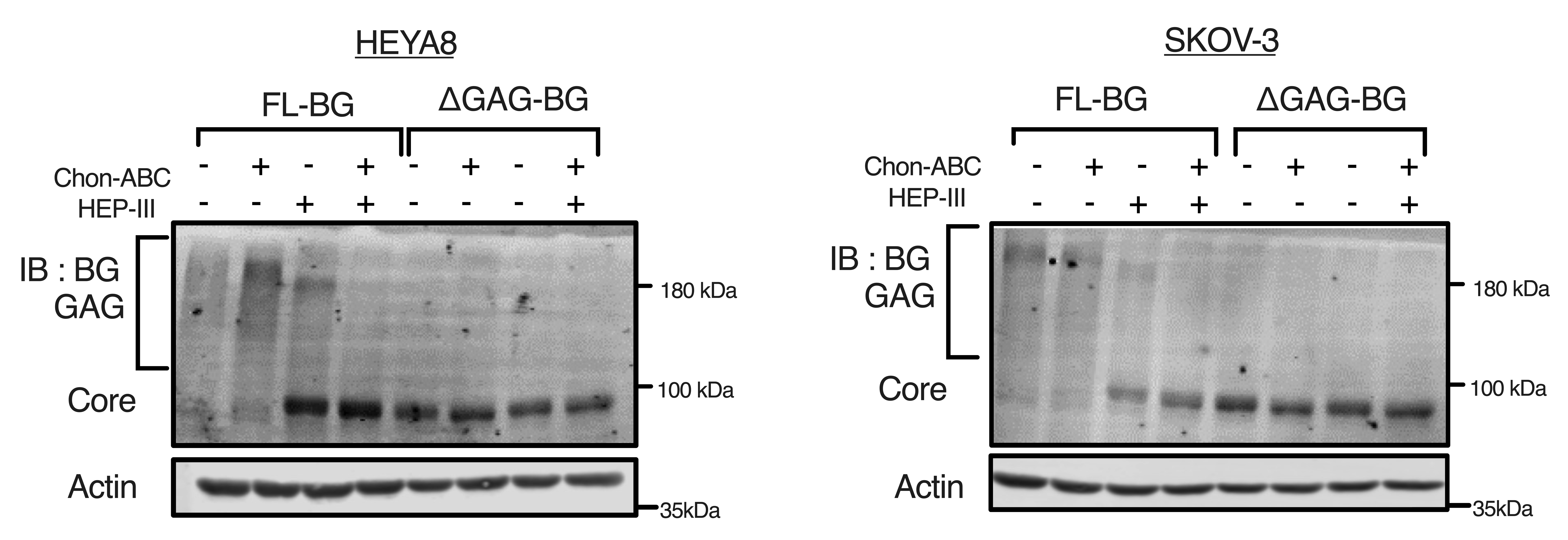

Supplement: Supplementary file 2 — Additional file 2: Supplementary Figure 2 [file 12964_2024_1496_MOESM2_ESM.tiff]

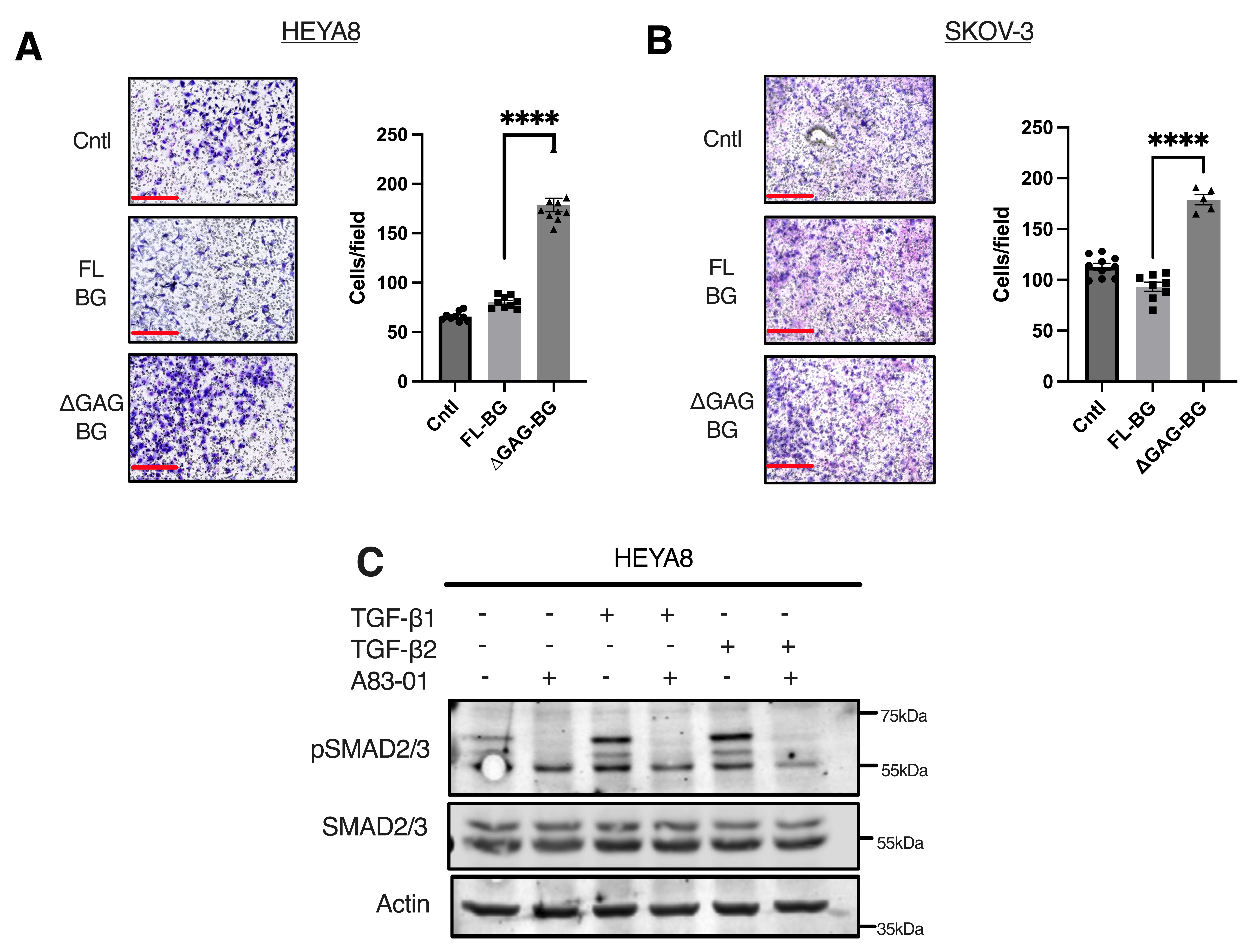

Supplement: Supplementary file 4 — Additional file 4: Supplementary Figure 4 [file 12964_2024_1496_MOESM4_ESM.tiff]

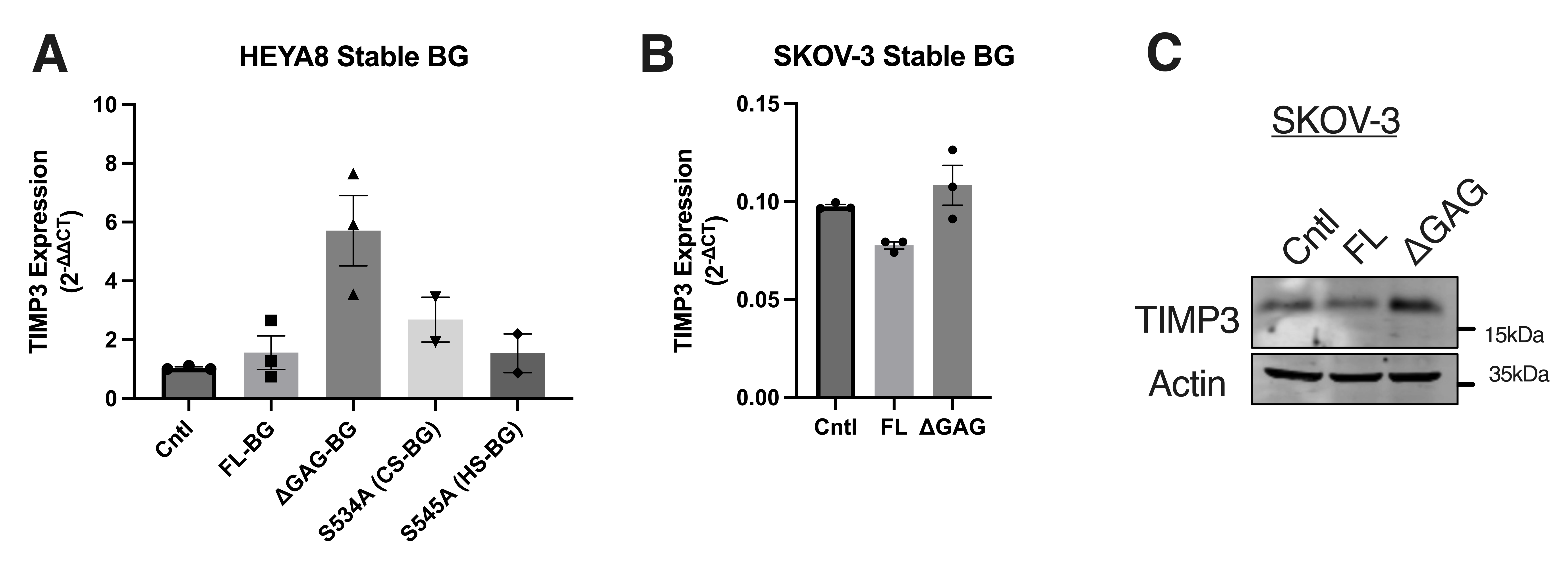

Supplement: Supplementary file 6 — Additional file 6: Supplementary Figure 5 [file 12964_2024_1496_MOESM6_ESM.tiff]
