# Supplementary material for: Glycosaminoglycan modifications of betaglycan regulate ectodomain shedding to fine-tune TGF-β signaling responses in ovarian cancer
Source: Cell Commun Signal. 2024 Feb 15;22:128. doi: 10.1186/s12964-024-01496-y (PMC10870443; doi:10.1186/s12964-024-01496-y)
Supplement: Supplementary file 5 — Additional file 5: Supplementary Table 1. Differentially expressed MMP or ECM-related genes from RNA-SEQ [file 12964_2024_1496_MOESM5_ESM.docx]

Supplementary Table 1

**Differentially expressed MMP or ECM-related genes from RNA-SEQ**

| **S545A (HS-BG) vs ∆GAG-BG** | **log_2_FC** | **-log10(pval)** |
| --- | --- | --- |
| *ADAMTS17* | 0.9533 | 1.5192 |
| *TMPRSS11CP* | 2.0375 | 1.7316 |
| *MMP3* | 2.3732 | 1.9306 |
| *TIMP3* | -0.5350 | 1.9352 |
| *PRSS48* | 1.0411 | 1.4261 |
| *PRSS36* | -0.9094 | 1.6709 |
| *ADAM8* | 0.5352 | 1.4499 |
| *ADAMTS3* | -0.5870 | 1.8941 |
| *MMP23B* | -1.5254 | 1.5052 |
| *TGFBR3* | -3.1818 | 2.0212 |
|  |  |  |
| **S534A (CS-BG) vs ∆GAG-BG** | **log_2_FC** | **-log10(pval)** |
| *TIMP3* | -1.0342 | 5.3645 |
| *ADAMTS9* | 1.3538 | 1.6960 |
| *TMPRSS11CP* | 2.1849 | 1.6229 |
| *TMPRSS6* | -2.3199 | 2.8800 |
| *ADAM12* | 0.6949 | 1.4706 |
| *TGFBR3* | -3.4550 | 2.0981 |
