# Supplementary material for: Glycosaminoglycan modifications of betaglycan regulate ectodomain shedding to fine-tune TGF-β signaling responses in ovarian cancer
Source: Cell Commun Signal. 2024 Feb 15;22:128. doi: 10.1186/s12964-024-01496-y (PMC10870443; doi:10.1186/s12964-024-01496-y)
Supplement: Supplementary file 7 — Additional file 7: Supplementary Table 2. Patient ascites fluid sample list [file 12964_2024_1496_MOESM7_ESM.docx]

Supplementary Table 2

**Patient ascites fluid sample list**

| **AF ID** | **Depository** | **Histology** | **Stage** | **Survival (Months)** | **sol-BG Concentration (pg/mL)** |
| --- | --- | --- | --- | --- | --- |
| DAF1 | DUKE | Endometroid | 2B | NA | 1192.1 |
| DAF2 | DUKE | Endometroid | 3 | NA | 875.2 |
| DAF3 | DUKE | mucinous | 3C | NA | 378.2 |
| DAF4 | DUKE | mucinous | 3C | NA | 1437.1 |
| DAF5 | DUKE | mucinous |  | NA | 1134.1 |
| DAF6 | DUKE | mucinous | 1A | NA | 810.2 |
| DAF7 | DUKE | serous | 3C | NA | 1523.1 |
| DAF8 | DUKE | serous | 3 | NA | 1509.6 |
| DAF9 | DUKE | serous | 4 | NA | 1543.6 |
| DAF10 | DUKE | serous | 4 | NA | 1281.7 |
| DAF11 | DUKE | serous | 3C | NA | 1027.8 |
| DAF12 | DUKE | serous | 4 | NA | 915.9 |
| DAF13 | DUKE | serous | 4 | NA | 1691.5 |
| DAF14 | DUKE | serous |  | NA | 551.0 |
| DAF15 | DUKE | serous | 3B | NA | 657.0 |
| DAF16 | DUKE | serous | 3C | NA | 943.0 |
| DAF17 | DUKE | serous | 4 | NA | 140.9 |
| DAF18 | DUKE | serous | 1C | NA | 696.0 |
| DAF19 | DUKE | serous | 3 | NA | 595.5 |
| DAF20 | DUKE | serous | 3 | NA | 910.7 |
| DAF21 | DUKE | serous | 3 | NA | 317.2 |
| DAF22 | DUKE | serous | 3 | NA | 505.2 |
| DAF23 | DUKE | serous | 3C | NA | 1255.6 |
| DAF24 | DUKE | serous | 3C | NA | 927.0 |
| DAF25 | DUKE | NA | NA | NA | 1872.5 |
| DAF26 | DUKE | NA | NA | NA | 482.1 |
| DAF27 | DUKE | NA | NA | NA | 847.3 |
| PAF1 | Penn. State | serous | 3C | NA | 1128.5 |
| PAF2 | Penn. State | granulosa | 1A | NA | 1057.2 |
| PAF3 | Penn. State | serous | 4 | NA | 1084.0 |
| PAF4 | Penn. State | serous | 4 | NA | 1067.5 |
| PAF5 | Penn. State | serous | 2C | NA | 1064.4 |
| PAF6 | Penn. State | serous | 4 | NA | 1096.4 |
| PAF7 | Penn. State | NA | NA | NA | 982.5 |
| UAF1 | UAB | papillary serous | 3C | 47 | 95.7 |
| UAF2 | UAB | serous adenocarcinoma | 3C | 88 | 174.5 |
| UAF3 | UAB | HGS | 3C | 32 | 210.0 |
| UAF4 | UAB | serous | 3C | 24 | 253.8 |
| UAF5 | UAB | papillary serous | 3C | 83 | 255.3 |
| UAF6 | UAB | Pap serous | 3C | 54 | 316.4 |
| UAF7 | UAB | papillary serous | 3C | 12 | 449.0 |
| UAF8 | UAB | NA | 3C | NA | 766.3 |
| UAF9 | UAB | HGS | 3C | 11 | 770.9 |
| UAF10 | UAB | Carcinosarcom | 4 | 33 | 847.6 |
| UAF11 | UAB | HGS | 3C | 23 | 866.3 |
| UAF12 | UAB | HGS | 3C | 3 | 903.5 |
| UAF13 | UAB | Adeno | 3C | 24 | 905.8 |
| UAF14 | UAB | serous | 3C | 51 | 920.1 |
| UAF15 | UAB | serous papilary | 3C | 24 | 1003.7 |
| UAF16 | UAB | Pap serous | 3C | 10 | 1006.6 |
| UAF17 | UAB | Prim Perit | 4 | 48 | 1063.7 |
| UAF18 | UAB | HGS | 3C | 53 | 1130.2 |
| UAF19 | UAB | ovary | 4 | 7 | 1131.5 |
| UAF20 | UAB | Prim Perit | 3C | 63 | 1162.2 |
| UAF21 | UAB | Prim Perit | 3C | 50 | 1164.0 |
| UAF22 | UAB | HGS | 3C | NA | 1569.8 |
| UAF23 | UAB | serous | 3C | 13 | 1579.9 |
| UAF24 | UAB | Endometroid | 4 | 13 | 1713.9 |
| UAF25 | UAB | Pap serous | 3C | 22 | 1791.1 |
| UAF26 | UAB | Pap serous | 3C | 22 | 2632.7 |
